# How Mechanical and Physicochemical Material Characteristics Influence Adipose-Derived Stem Cell Fate

**DOI:** 10.3390/ijms24043551

**Published:** 2023-02-10

**Authors:** Svenja Nellinger, Petra Juliane Kluger

**Affiliations:** 1 Reutlingen Research Institute, Reutlingen University, 72762 Reutlingen, Germany; 2 School of Life Sciences, Reutlingen University, 72762 Reutlingen, Germany

**Keywords:** adipose-derived stem cells, biomaterials, stiffness, topography, modification

## Abstract

Adipose-derived stem cells (ASCs) are a subpopulation of mesenchymal stem cells. Compared to bone marrow-derived stem cells, they can be harvested with minimal invasiveness. ASCs can be easily expanded and were shown to be able to differentiate into several clinically relevant cell types. Therefore, this cell type represents a promising component in various tissue engineering and medical approaches (e.g., cell therapy). In vivo cells are surrounded by the extracellular matrix (ECM) that provides a wide range of tissue-specific physical and chemical cues, such as stiffness, topography, and chemical composition. Cells can sense the characteristics of their ECM and respond to them in a specific cellular behavior (e.g., proliferation or differentiation). Thus, in vitro biomaterial properties represent an important tool to control ASCs behavior. In this review, we give an overview of the current research in the mechanosensing of ASCs and current studies investigating the impact of material stiffens, topography, and chemical modification on ASC behavior. Additionally, we outline the use of natural ECM as a biomaterial and its interaction with ASCs regarding cellular behavior.

## 1. Introduction

Cells continuously interact at a high level with their surrounding extracellular matrix (ECM) in vivo and growth substrates or scaffolds in vitro and modulate their functional phenotypes to maintain homeostasis [1,2,3]. The surrounding material can provide cues that control migration, adhesion, proliferation, and even differentiation of the cells. These cues include physicochemical properties, such as surface topography, chemical modifications and composition, and mechanical properties. Next to the used biomaterial, the choice of a suitable cell source is crucial for success in tissue engineering approaches. Adipose-derived stem cells (ASC), a subpopulation of mesenchymal stem cells (MSC), represent a promising cell source regarding several tissue engineering aims. In contrast to the widely used bone marrow-derived MSCs, they can be isolated in higher numbers with less invasive techniques and can be differentiated into different cell-lineages, which are in strong demand for tissue engineering approaches [4,5]. In the context of cell-based therapies and tissue engineering, it is essential to understand how the physicochemical properties of material surfaces influence ASC behavior. In the last years, a great effort was made to investigate the interactions on the cell–material interface. Understanding these interactions would be a milestone in medical engineering and regenerative medicine by providing specific material designs, which would facilitate and promote tissue repair and regeneration. For tissue engineering, the challenge is to create an in vitro matrix, which promotes progenitor cell migration, adhesion, and proliferation and induces differentiation, extracellular matrix synthesis, and integration with host tissue. Therefore, the major approaches in developing new biomaterials are mimicking certain advantageous characteristics of the natural ECM of the specific cells. This review gives an overview of the current findings of ASC–material interaction, especially regarding the influence of physical and chemical properties and surface topography on the adhesion, proliferation, and differentiation of ASCs. The last part summarizes the current studies using natural ECM as a biomaterial and its impact on ASC behavior.

## 2. Adipose-Derived Stem Cells

MSCs are multipotent stem cells that can differentiate into multiple cell types of the mesoderm, such as chondrocytes, osteoblasts, and adipocytes, and non-mesenchymal cell lines, such as neuronal cells, cardiac cells, and skeletal muscle cells. MSCs can be isolated from a wide range of tissues including bone marrow, umbilical cord stroma, and adipose tissue [6]. They exhibit a spindle-shaped fibroblast-like morphology. According to the Minimal Criteria of the International Society of Cellular Therapy, MSCs have to be plastic adhered, exhibit tri-lineage differentiation potential (adipogenic, osteogenic, and chondrogenic), and express cluster of differentiation (CD)73, CD90 and CD105 [7].

ASCs are part of the stromal vascular fraction (SVF) of adipose tissue from where they can be isolated by enzymatic digestion [8]. The SVF of a tissue describes the entirety of all cells of blood vessels and stroma [9,10]. Despite defining ASCs is still a challenge, CD13, CD29, and CD44 count as the unofficial markers for ASCs [11]. Compared to widely used bone marrow-derived MSCs, ASCs have several advantages. For example, they exhibit a 2500-fold higher abundance and a higher differentiation and proliferation potential than bone marrow-derived MSCs. Furthermore, adipose tissue harvest is cheaper, safer, and less invasive compared to bone marrow aspiration [12,13]. However, also ASCs have some limitations regarding tissue harvesting. There is evidence that local anesthetic agents might negatively impact ADSC viability and quantity [14]. Furthermore, liposuction in summer should be avoided due to the risk of infections. Contrary, isolation from whole adipose tissue can be performed every time.

Cultured ASCs secret various immunomodulatory cytokines and growth factors that are relevant for cell therapy [15,16]. It can be suggested that when ASCs are transplanted into inflammatory regions, they actively secret these factors and significantly promote wound healing and tissue repair. This may make ASCs a powerful tool for use in future approaches in the development of cell- and tissue-based therapeutics. To date, there are several clinical trials in several research areas investigating the therapeutic potential of ASCs [17,18,19,20,21,22,23]. However, several points remain unclear. For example, the dependence of differentiation potential from the donor’s age, gender, and anatomic location of the fat source [24]. Despite these limitations, ASCs represent a promising cell source for adipose tissue engineering and regenerative medicine.

**Figure 1:**
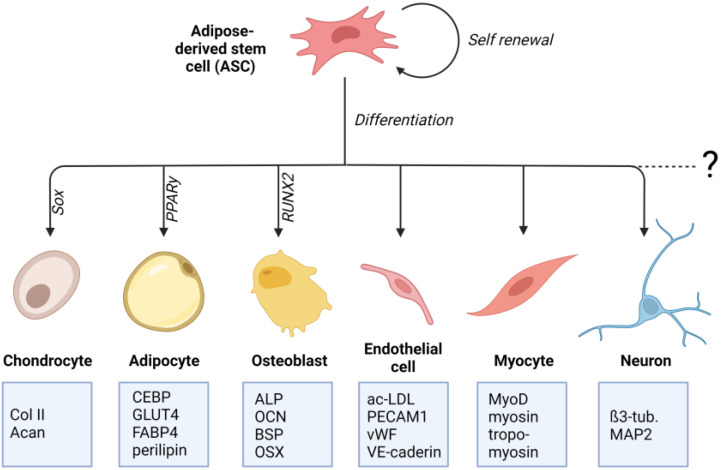
Differentiation potential of adipose-derived stem cells (ASCs): ASCs are multipotent mesenchymal stem cells that can be isolated from adipose tissue. ASCs can differentiate into mesenchymal cell lines such as osteocytes, adipocytes, and chondrocytes. There is also evidence for their differentiating potential into non-mesenchymal cell lines, such as endothelial cells, neuronal cells, and cardiac and skeletal myocytes. For each differentiation lineage, a specific “master transcriptional regulator” is identified. For adipogenic lineage it is PPARγ, for osteogenic lineage it is RUNX2, and for chondrogenic lineage it is Sox9. Lineage-specific differentiation can be determined by specific proteins that are listed in the boxes (created with BioRender.com).

Crucial for the determination to differentiate into a particular cell type is the specific differentiation signals received by the cell. These signals can either be soluble factors in the cell culture medium or mechanical signals transmitted through matrix properties. The differentiation process involves a complex and highly orchestrated regulation of the expression of lineage-specific transcription factors (Figure 1). These “master transcriptional regulators” are PPARγ for adipogenic lineage [25], RUNX2 for osteogenic lineage [26], and Sox9 for chondrogenic lineage [27]. Lineage-specific differentiation can be determined by specific proteins. For chondrogenic differentiation, these include the important ECM genes Col2a1 and Acan, Col9a1, Col27a1, and Matn1 [28,29]. Adipogenic differentiation can be proven by proteins involved in insulin sensitivity, lipogenesis, and lipolysis, including fatty acid synthase (FAS), glucose transporter type 4 (GLUT4), lipoprotein lipase (LPL), fatty acid binding protein 4 (FABP4), perilipin, and adipokines [30,31,32,33,34]. Classical osteogenic markers include proteins, such as collagen type I, alkaline phosphatase (ALP), osteocalcin (OCN), and bone sialoprotein (BSP) [35]. Another important transcription factor involved in regulating osteogenic differentiation is the zinc-finger transcription factor osterix (OSX) [36].

## 3. Signaling Pathways of Cell-Matrix/Cell-Material Interaction

**Figure 2:**
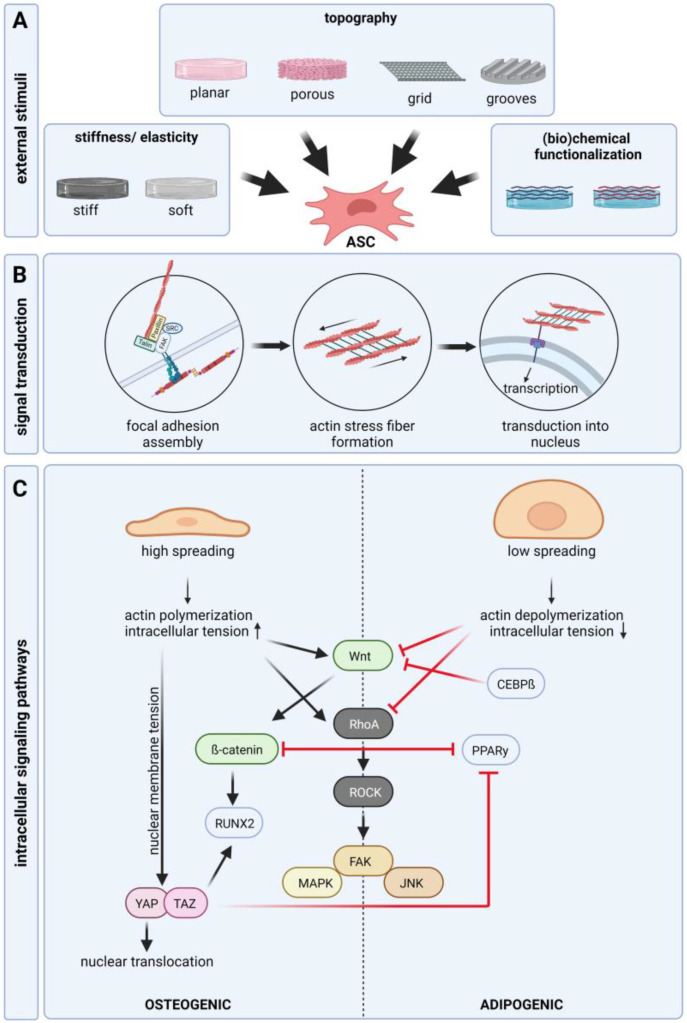
Impact of material properties on the ASC behavior and involved signaling pathways. (**A**): Material properties, such as stiffness/elasticity, pore size/porosity, topography/geometry, and (bio)chemical functionalization (and subsequent protein adsorption) have a strong impact on ASC behavior by influencing cytoskeletal reorganization, integrin expression, and focal adhesion assembly. (**B**): Focal adhesion assembly that is induced by ECM interaction is transduced into the nucleus via stress fiber formation and their interaction with the nuclear membrane. Signal transduction into the nucleus impacts the activity of transcription factors, histone modification, epigenetics, and chromosome condensation. (**C**): Enhanced spreading of the cell leads to actin polymerization and an increase of intracellular tension and subsequent activation of osteoinductive signaling pathways Wnt/β-catenin, RhoA/ROCK, and YAP/TAZ. In contrast, reduced spreading leads to decreased intracellular tension and inhibition of osteoinductive signaling pathways which leads to adipogenic differentiation (created with BioRender.com).

In all tissues, the occurring cells have close structural and functional connections to the surrounding extra cellular matrix (ECM)—a highly complex network of different protein fibers (collagen, elastin, reticular fibers), proteoglycans, and adhesion molecules filling the interspace between the cells. The ECM forms a three-dimensional scaffold that provides mechanical stability to the tissue. Adhesion is necessary for the expansion and differentiation of adhered cells, such as ASCs. It is well known that cells sense and response to the physical and chemical properties of their environment, which mostly results in the up- or downregulation of specific intercellular signaling pathways. These pathways control and modulate cellular behavior, such as adhesion, migration, proliferation, and differentiation. At the interface to materials, the cells interact mainly with the adsorbed proteins occurring in the surrounding culture medium or biologic fluids e.g., serum or blood. Different proteins exhibit different adsorption dynamics and further surface properties, such as topography and surface chemistry, change the absorption ability of proteins on the material. Therefore, the composition of the protein layer on the material and the resulting interaction with cells depends on the combination of the protein composition in the surrounding fluid and the physicochemical properties of the material itself (Figure 2A). The first binding of the cell, mediated by physical and chemical forces, is followed by receptor-mediated adhesion. These adhesion receptors are transmembrane receptors, such as integrins. Integrins are heterodimers consisting of an α and a β subunit. With its extracellular part, integrin binds to matrix proteins, including fibronectin or laminin, whereas the intracellular part binds to other proteins, such as paxillin and vinculin, forming focal adhesion contacts and interacting with the f-actin of the cytoskeleton. The interaction of integrin and matrix proteins is mainly mediated by specific amino acid sequences (e.g., arginine–glycine–aspartic acid; RGD) in proteins, for example, fibronectin and vitronectin [37]. It could be shown that activation of specific integrin subtypes by binding to matrix proteins is essential for cell fate [38]. For ASCs several studies showed an expression of integrin β1 and fibronectin binding integrin α4 and integrin α [9,39]. Sun et al. demonstrated that integrin α5 acts as a major regulator of osteogenic differentiation [40]. However, some discrepancies exist, which may trace back to differences in cell isolation and culture. Further, the part of the body from where the tissue was taken and the age of the donor may influence the surface protein expression profile. Different studies revealed an altered integrin expression during the differentiation of ASCs and the overexpression of specific integrins leads to the suppression of differentiation [41]. For adipogenic differentiation studies found a downregulation of α5 but an upregulation of α6 [41,42]. In line with this, studies found that the upregulation of α5 increases osteogenic differentiation [43,44].

Integrin binding and **focal adhesion assembly** induce actin polymerization and the generation of contractility of **actin stress fibers** (Figure 2B). Via focal adhesion and integrin-ECM binding, the stress fibers are in contact with the extracellular environment. A higher density of extracellular integrin ligands leads to the formation of larger focal adhesions and subsequent cellular spreading [45]. Stress fibers are actomyosin structures composed of crosslinked F-actin and myosin-2. Force generated by actomyosin contraction determines the shape of the cell, which affects cellular behavior. Cell contraction creates traction force by pulling the surface trough integrin binding sites leading to structural deformation of the ECM depending on stiffness. Its ECM supports this force and stronger integrin signaling is induced maintaining a positive feedback loop [46]. **Nuclear mechanosensation** is a key process in response to physical stimuli. Stress fibers are connected to the nuclear membrane, which enables the transmission of external forces or cytoskeletal tension, causing structural deformation of the nucleus [47]. Mechanical stresses can increase the tension of the nuclear membrane, the nuclear import and activity of transcription factors, histone modification, epigenetics, and chromosome condensation. Changes in the external environment, such as substrate stiffness, can regulate the expression of nuclear proteins that are strongly correlated with tissue-stiffness-specific stem cell differentiation [48].

Matrix-induced integrin-mediated intracellular tension and cell morphology are closely linked to cell fate. Osteogenesis is favored when cells can spread over a large area, and adipogenesis is promoted when cell spreading is impaired (Figure 2C) [49,50,51]. However, mechanical regulation of stem cell differentiation requires a certain level of intracellular tension. If the ligand density is too low, differentiation is suppressed regardless of the spreading of the cell [52]. Through the focal adhesion kinase, a tyrosine kinase that is activated in response to ligation of α and β integrin-subunits, focal adhesion contacts and integrin binding is connected **to several intracellular signaling pathways**, such as the Rho A/ROCK pathway, MAP kinase pathway, and Wnt/β-catenin pathway [53,54,55,56]. Several studies demonstrated that suppression of WNT/β-catenin and RhoA/ROCK signaling is essential for adipogenic differentiation [57,58,59,60]. In contrast, it is well shown that activation of the WNT/β-catenin and RhoA/ROCK pathway is important for osteogenic differentiation. Cellular morphology directly mediates **RhoA activity,** which leads to the activation of ROCK. Wide spreading of the cell and high integrin signaling activates ROCK creating intracellular tension by phosphorylation of myosin and initiating actin polymerization. ROCK further initiates the activation of downstream signaling pathways (JNK, MAPK, and FAK) associated with osteogenic differentiation [61]. Reduced intracellular tension inhibits RhoA activity and downstream signaling, which favors the adipogenic differentiation od ASCs. In the absence of **Wnt signaling, β-catenin** is continuously degraded. After activation of Wnt signaling, β-catenin accumulates and is translocated to the nucleus where is acts as a transcription factor and increases RUNX2 expression. Nuclear translocation of β-catenin is also enhanced by substrate stiffness, which induces osteogenic differentiation [62,63,64,65]. In addition to acting as an osteoinductive transcription factor, β-catenin acts as an inhibitor of adipogenic transcription factor PPARγ. Activation of β-catenin leads to the degradation of PPARγ and vice versa [66]. Furthermore, adipogenic transcription factor CEBPβ was shown to inhibit Wnt signaling [67]. However, the regulation of osteogenic differentiation through Wnt signaling seems to be a matter of timing [68,69]. Thus, the expression pattern of integrins and focal adhesion assembly plays a pivotal role in regulating cell behavior by the induction of cytoskeletal reorganization and several intracellular signaling pathways regulating morphology, adherence, proliferation, and differentiation [70,71]. Another signaling pathway involved in stem cell differentiation is YAP/TAZ. TAZ acts as a coactivator of RUNX2 while inhibiting PPARγ [72]. Translocation of YAP into the nucleus was shown to increase in response to physical stimuli. Mechanical stress applied to the nucleus increases nuclear membrane tension and expands nuclear pores, thereby promoting the inflow of YAP [73].

## 4. Influence of Material Properties on Adipose-Derived Stem Cell Fate

**Figure 3:**
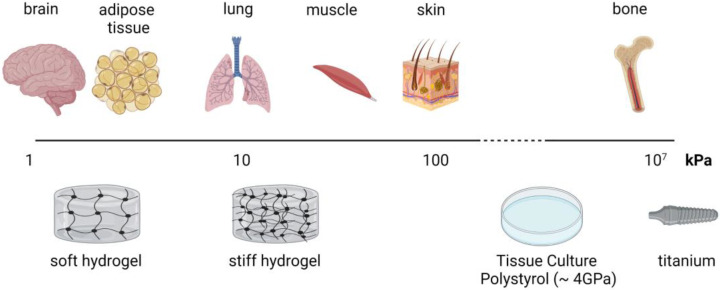
Overview of the stiffness of different tissue and common cell culture and implant materials (created with BioRender.com).

Next to the generally used soluble biochemical factors, the physical properties of the material the cells are exposed to play an important role in defining stem cell fate. Various methods have been used to determine cellular functions by changing cell adhesion through changes in the external substrates using micro- and nanosized topographies (e.g., pores and patterns), alterations in stiffness, and chemical modification. The cells sense the properties of their surrounding substrata and respond to them in various ways, including cytoskeletal reorganization, altered integrin expression, focal adhesion assembly, and epigenetic alterations. These effects further influence adhesion, differentiation, and proliferation (Figure 3).

### 4.1. Stiffness

The stiffness of a material is related to the loads (forces exerted on the material) and deformation (changes in shape). As it is independent of the structure, Young’s modulus is one of the most common measures of (bio)material stiffness. Materials that are stiffer and do not deform as easily exhibit a high Young’s modulus [74]. Cells of different tissues are surrounded by microenvironments with a wide range of mechanical stiffness. For example, Young’s modulus of adipose tissue is ~3 kPa and of bone tissue is ~10–20 GPa [75,76]. Classical cell culture materials do not fit the range of stiffness of soft tissues, such as adipose tissue or the brain. For the culture of the cells in a soft tissue-like environment, one has to use hydrogel materials. The most common hydrogel materials include alginate, gelatin, polyacrylamide, collagen, and PDMS. An upcoming promising hydrogel material for adipose tissue engineering is gellan gum [77,78,79]. Under pathophysiological conditions, tissue can exhibit altered mechanical properties. For example, tumor stroma is shown to be 5–20 times stiffer than healthy tissue [76,80].

The suggestion that mechanical cues play an essential role in tissue development is not new. In the late 1800s, Julius Wolff proposed that mechanical stresses play a critical role in normal bone development and adaption. A variety of current studies show that next to biochemical stimuli, biophysical cues, such as the stiffness of a substrate or matrix, plays an essential role in regulating ASC behavior. Several studies demonstrate that softer substrates enhance neuronal and adipogenic differentiation of ASCs, while increasingly stiffer substrates enhance myogenic, chondrogenic, and osteogenic differentiation (Table 1). Considering the influence on cell morphology a quite uniform picture emerges: on soft surfaces, spreading is decreased and cells exhibit a rounded shape, whereas on rigid surfaces ASCs exhibit more spreading and polygonal shapes. A similar picture can be observed considering proliferation: on soft materials, ASC proliferation is enhanced, whereas on rigid materials proliferation is decreased. Generally, it is difficult to compare the given stiffness in various publications as the values can vary depending on the method used for determination. In the last years, there has been a great effort to introduce standardized methods for the determination of the stiffness of tissue-engineered products, e.g., ASTM standards (ASTM F561-19, ASTM F2150-13, and ASTM D638-14) [76]. In this review, the values stated in the references are given for orientation. Furthermore, chemical characteristics of the material (e.g., functional groups and charge) might have an impact on ASC behavior that might cover the effect of the stiffness. As there was a wide range of different materials used in the studies comparison of the results is complicated.

To evaluate the influence of matrix stiffness on differentiation it has to be distinguished between studies using differentiation factors in culture medium and studies only using growth medium to investigate the influence of stiffness. It can be assumed that in studies using soluble differentiation factors, the differentiation was mainly induced by the chemical cues in the culture medium, which may cover the effects of the stiffness of the surrounding material. Major et al. found increased CEBPα expression in ASCs cultured on softer substrates (PPARγ was not affected) and upregulation of osteogenic genes RUNX2 and ALP [81]. They further investigated the combined effects of matrix stiffness (3 kPa and 35 kPa), shape (circle, rectangle, and square), and size (1000; 5000; 10,000 cm^2^) on adipogenic and osteogenic differentiation of ASCs. In this comprehensive study, they were not able to detect clear trends for the expression of specific differentiation markers depending on the investigated characteristics. Only for RUNX2, they found an increase of expression with increasing size on the 3 kPa matrix for all shapes. Shridhar et al. and Xie et al. found adipogenic differentiation on rather stiff substrates (36 kPa and 46 kPa), which is much more stiff than native adipose tissue [62,82]. This indicated that the soluble factors forced the ASC to differentiate into the adipogenic lineage instead of the matrix stiffness. Shridhar et al. used decellularized adipose tissue as a cultured substrate. Natural ECM is known to induce tissue-specific differentiation of stem cells by providing a combination of the tissue-specific composition of structural and chemical cues. Investigation of chondrogenic differentiation in the context of matrix stiffness is rare. This might be because classically chondrogenic differentiation protocols prescribe chondrogenic differentiation in pellets or spheroids. In this constellation, the cell–cell interaction is higher than the cell–matrix interaction and might diminish the effect of matrix stiffness. Teong et al. and Zigon-Branc et al. found chondrogenic differentiation of ASCs in hydrogels with a stiffness of 0.5–8 kPa with an upregulation of aggrecan and collagen type II [83,84]. For chondrogenic inducer SOX9, there are inconsistent results. A variety of studies found osteogenic differentiation on substrates with stiffness ranging from 7 to 1000 kPa. The ELP used in the study of Gurumurhy et al. gained interest as a material for wound dressing [85] indicating its use for soft tissues [86]. Newman et al. (3–5 kPa) and Betre et al. demonstrated adipogenic and chondrogenic differentiation of ASCs cultured in ELP materials [87,88].

Much more conclusive are studies investigating the effect of matrix stiffness without supplementation of soluble differentiation factors in culture medium. For these studies, a stiffness-depended differentiation can be observed: adipogenic differentiation at 0.5–5 kPa and osteogenic differentiation at 8.5–4500 kPa. Kim et al. investigated the differentiation of ASCs on GelMA hydrogels with stiffness gradients ranging from 3.5 kPa to 13 kPa [89]. They found a decrease in PPARγ expression with an increase in stiffness and a peak of MyoD expression at 10 kPa. Banks et al. found that the expression of osteogenic genes OCN, Col I, and ALP of ASCs cultured without factors in the medium are significant elevated at a polyacrylamide hydrogel stiffness of 37 kPa compared to 14 kPa and 5 kPa [90]. Further, they showed that osteogen-specific alkaline phosphatase activity significantly increases with increasing substrate stiffness. Conversely, adipogenic specific transcription factor PPARγ decreases with increasing stiffness. The working group of Guneta et al. obtained similar results [91]. They observed adipogenic differentiation at 3.5 kPa. However, they found osteogenic differentiation at 13 kPa, which is lower compared to Banks et al. This effect might occur due to other material characteristics, such as different pore sizes and porosity of the used AlgiMatrix^®^. Regarding adipogenic differentiation, these findings are in line with Young et al. who demonstrated adipogenic differentiation of ASCs on substrate stiffness of 2 kPa. Lee et al. compared ASCs and BM-MSCs regarding their response to different matrix stiffness [92]. They found that softer polyacrylamide hydrogels (0.5 kPa) lead to elevated Nile red staining intensity and high accumulation of lipid droplets in ASCs. In contrast, PPARγ expression as a marker for adipogenic differentiation is less influenced by substrate stiffness. Furthermore, they demonstrated that the influence of matrix stiffness is higher in BM-MSCs than in ASCs and in the absence of media supplements adipogenesis of ASCs is not significantly influenced by matrix stiffness. As there is a wide range of materials used for tissue engineering approaches it is difficult to compare different studies using different materials and culture parameters. Only a few studies investigated all three mesenchymal lineage makers. However, these studies draw a consistent picture of the induction of lineage-specific differentiation by substrate stiffness.

It has to be considered that the obtained results depend on the investigated differentiation markers. For example, in adipogenic differentiation, early markers are the transcription factors PPARγ and C/EBP, whereas lipid droplets occur at a later time point of differentiation. Assuming that matrix properties induce a differentiation, this process would take more time compared to medium-induced differentiation. Therefore, early markers, such as transcription factors may represent more dependable evidence. Also, the impact of the used batch of FCS in the growth medium should be considered. It is well known that different batches of FCS have a different impact on cellular behavior, including differentiation.

Due to the important role of integrin-mediated adhesion to the ECM in regulating cell behavior, different stiffness may lead to different cellular signals regulating differentiation. These pathways, which are known to regulate stem cell behavior, are the ERK-MAPK pathway, Wnt/β-catenin pathway, and RhoA/Rock pathway. Activation of these pathways seems to be connected with osteogenic differentiation, whereas inhibition may lead to adipogenic differentiation. An altered integrin expression caused by substrate stiffness may lead to activation or inhibition of the pathways resulting in osteogenic or adipogenic differentiation. As the studies show, different substrate stiffness allow altered spreading and subsequent changes in morphology. Zhang et al. demonstrated the downregulation of proteins of the Wnt/β-catenin pathway (β-catenin, cyclin-D, Lef-1) and RhoA/Rock pathway in ASCs cultured on softer materials and upregulation in ASCs cultured on stiffer materials [93]. Additionally, Xie et al. found the same expression pattern for β-catenin [62]. Further, there is evidence that the mechanical stiffness of the substrate influences the sensitivity of cells towards other exogenous factors, such as growth or differentiation factors in the culture medium [94,95,96,97].

Materials with similar stiffness to the native tissue enhance differentiation into the respective lineage. Soft material was found to favor adipogenic differentiation, whereas stiff materials favor osteogenic differentiation. For medium-range stiffness, enhancement of chondrogenic differentiation was shown.

**Table 1:** Overview of the influence of substrate stiffness on ASC differentiation, adhesion, morphology, and proliferation. (PDMS: polydimethylsiloxane; ELP: elastin-like polypeptide; PTFE: polytetrafluoroethylene; PVA: polyvinyl alcohol; GO: graphene oxide; PEEU: polyetheresterurethane; ECM: extracellular matrix).

Differentiation	Stiffness (kPa)	Soluble Factors	Material		Results	Ref.
Adipogenic	1.4–6	+	PDMS	2D	Spreading ↓, disorganized actin filaments, Oil Red O ↑, Rho A ↓, ROCK 1/2 ↓, proteins of Wnt/β-catenin pathway ↓	[93]
	0.5	−	Polyacrylamide	2D	Spreading ↓, PPARγ n.a., Nile Red ↑ Neuronal: β3 tubulin ↑, MAP2 ↑	[92]
	2	−	Adipose ECM functionalized polyacrylamide	2D	Rounded shape, spreading ↓, PPARγ ↑, CEBP ↑, ap2 ↑	[98]
	3	+	Polyacrylamide	2D	CEBPα ↑	[81]
	~4	−	GelMA	2D	PPARγ ↑	[89]
	5	−	Polyacrylamide	2D	Oil Red O ↑	[90]
	36	+	Decellularized adipose tissue	2D	Proliferation ↑, PPAR γ n.a., LPL ↑, adiponectin ↑; PLIN ↑, perilipin ↑	[82]
	46	+	PDMS	2D	Spreading ↓, β-catenin ↓, Oil Red O ↑, PPARγ ↑, CEBPα ↑	[62]
Myogenic	~12	−	GelMA	2D	MyoD ↑	[89]
Chondrogenic	8	+	Methacylated hyaluronan	3D	Aggrecan ↑, collagen type II ↑, SOX9 ↓	[83]
Osteogenic	35	+	Polyacrylamide	2D	RUNX2 ↑, ALP ↑	[81]
	37	−	Polyacrylamide	2D	ALP ↑, COL1A1 ↑, OCN ↑	[90]
	53.6–134	+	PDMS	2D	Spreading ↑, polygonal shape, bundled actin fibers, Alizarin Red ↑, Rho A ↑, Rock 1/2 ↑, proteins of Wnt/β-catenin pathway ↑	[93]
	61.8	+	ELP-collagen	3D	ALP ↑, osteocalcin ↑, Alizarin Red ↑	[86]
	660	+	PTFE/PVA(/GO)	2D	Alizarin Red ↑, ALP ↑, RUNX2 ↑, osteoclacin ↑, osteonectin ↑	[99]
	1000	+	PDMS	2D	Spreading ↑, β-catenin ↑, ALP ↑, RUNX ↑, OSX ↑	[62]
	4500	−	Electrospun PEEU	2D	Osteocalcin ↑, ALP ↑, hydroxyapatite ↑	[100]

### 4.2. Topography, Geometry, and Pore Size

The ECM of different tissues provides different structures in the micro- and nanometer range, in which the cells perceive and react in a specific manner. By mimicking the nanostructures of the natural ECM, nano-topography, such as grooves, pits, fibers, and tubes, can serve as a cell-stimulating cues to affect the regulation of ASC differentiation. Concerning the adhesion of ASCs on different surface topographies, there is broad consensus that, on rough surfaces, adhesion is enhanced compared to flat substrates [101,102,103]. On planar surfaces, adherent cells generally spread. Nanotopographical structures inhibit the spreading of the cells by decreasing the available adhesion sites and subsequent decreasing of focal adhesions. For example, Yun et al. demonstrated an altered focal adhesion assembly in ASCs cultured on nanopillars of different sizes. They found a decreased area of focal adhesions on pillars with a higher diameter, which leads to a higher spreading of the cells [104]. Yim et al. found that on nanogrooves ASCs exhibit lower focal adhesion complexes and actin filament level, which mediates cell adhesion. However, the adhesion rate on nano-structured PS was higher than on a flat substrate. They further found that integrin expression on patterned substrates is decreased compared to nonpatterned substrates [105]. Mobasserie et al. demonstrated that the shape of the nanogrooves is crucial for adhesion and proliferation. They found that on grooves with sloped walls, cell adhesion is enhanced compared to square-shaped and v-shaped grooves. Additionally, they found that on v-shaped grooves, cell proliferation is higher than on the other grooves [106]. Nano-topography is also known to influence cell morphology through contact guidance depending on cell type and structure geometry. A variety of studies demonstrate that on nanogrooves, ASCs exhibit a spindle-like morphology and align to the groove orientation, whereas on flat substrates they exhibit a more spread morphology and not aligned growth. After adhesion, integrins activate various protein tyrosine kinases (e.g., focal adhesion kinase, src, alb), serine-threonine kinases (e.g., mitogen-activated protein kinase (MAPK) and protein kinase C), and GTPases of the Rho-family [53,55,107]. As mentioned above, it seems that RhoA activation plays a pivotal role in ASC differentiation by mediating the assembly of actin stress fibers in response to extracellular stimuli in the interplay with its downstream effector Rho-associated protein kinase (ROCK) [108,109]. One possible mechanism by which extracellular cues regulate stem cell differentiation involves the extracellular signal-regulated kinase (ERK)—MAPK pathway [110]. The inhibition of RhoA reduces ERK activity resulting in adipogenic differentiation and expression of PPARγ, whereas active RhoA enhanced it resulting in osteogenic differentiation [110,111]. Several studies showed that on structured surfaces ASCs are orientated in a specific manner. For example, on grooves or fibers, the ASCs adopt a spindle-like morphology and are orientated with the structure or at a specific angle to the structure. As mentioned, a spindle-like morphology leads to the activation of RhoA, which in turn activates the ERK-MAPK pathway. The MAPK pathway is known to regulate the proliferation and differentiation of osteoblasts and osteoprogenitor cells [111,112,113].

#### 4.2.1. Nanogrooves and Nanofibers

A widely used and relatively simple nanostructure is nanogrooves or nanofibers applied on different substrates. Table 2 gives an overview of studies investigating the effect of grooves or fibers on ASC behavior. For this type of topography, only studies investigating osteogenic, myogenic, endothelial, neuronal, and tenogenic differentiation were found. No studies were found investigating adipogenic or chondrogenic differentiation. This might be due to the natural appearance of adipose tissue and cartilage, which do not exhibit the dominant fibrous/elongated structures compared to the other types of tissue. Further, it is known that fibrous/grooved structures lead to elongated morphologies activating the Wnt/β-catenin pathway and inhibiting adipogenic differentiation.

Five of the studies found an enhancement of osteogenic differentiation of cells cultured on aligned and random fiber structures. This effect can be observed with different materials, suggesting that topography is one of the main reasons. However, in four of these studies, differentiation was induced by soluble factors diminishing the influence of nanostructure. Calejo et al. investigated the ASC behavior on isotropic and anisotropic electrospun yarns. They demonstrated that ASCs cultured on isotropic fibers exhibited a higher ALP activity and Alizarin Red staining compared to ASCs on anisotropic fibers without the use of biochemical stimuli [114]. In a previous study from 2019, they showed osteogenic differentiation of ASCs on isotropic electrospun fibers modified with hydroxyapatite [115]. Hydroxyapatite exhibits similar chemical and structural characteristics as the inorganic bone components and, as it is known to have osteoinductive properties, it is a commonly used material for bone tissue engineering.

In the study of Ko et al. tissue sections of tendons were used as culture substrate, which may provide other natural signals, next to the topography, from the natural ECM influencing the cells [116]. Interestingly, they found no effect on RUNX2 expression indicating more osteochondral differentiation than osteogenic differentiation. Unfortunately, the expression of SOX9 as a chondrogenic marker was not investigated in this study. The supportive effect of ECM proteins was underlined by the findings of Chen et al. [117]. They cultured ASCs on randomly orientated electrospun PLGA/PLC fibers and found an upregulation of OCN, RUNX2, and OSX, whereas ALP activity and Col I secretion were not affected. These two factors were only elevated on fibers containing collagen I. Xue et al. investigated the differentiation potential of ASCs, BM-MSCs, and UC-MSCs on electrospun PCL nanofibers [118]. They demonstrated successful osteogenic differentiation of all three cell tapes with the highest potential in BM-MSCs, indicating an MSC source dependency for differentiation capacity. They found that in MSCs cultured on PCL nanofiber scaffolds expression of β-catenin is elevated. There is evidence that the activation of Wnt/β- catenin signaling induces osteogenic and suppresses adipogenic differentiation of ASCs via ROCK-mediated changes in the cytoskeleton, which up-regulates RUNX2 gene expression and suppresses PPARγ gene expression.

For endothelial and neuronal differentiation, grooves and structured network patterns are used instead of fibers. Shi et al. and Kim et al. demonstrated that nanogrooves enhance the endothelial differentiation of ASCs [119,120]. In the study of Kim et al., a complex method for generating sinusoidal grooves with different scales that cross each other was described as representing the natural shape of vascular structures. In general, fibers are more likely used for osteogenic, myogenic, and tenogenic differentiation, whereas grooves are used for endothelial or neurogenic differentiation. These structures tend to represent the natural shape or environment of the cells.

**Table 2:** Overview of the influence of grooved and nanofiber topography on ASC differentiation. (PDA: polydopamine, PLGA: polylactide-co-glycolide (PLGA), PCL: poly caprolactone, PU: polycarbonate-urethane).

Differentiation	Topography	Soluble Factors	Material		Results	Ref.
Osteogenic	Aligned fibers	+	Tendon	2D	Focal adhesion ↑, RUNX2 n.a., OPN ↑, COL I ↑, bone regeneration ↑	[116]
	Line patterns	+	Graphene oxid	2D	Spreading ↑, Alizarin Red ↑, ALP ↑, OCN ↑	[121]
	Fibers random	+	PLGA/PLC collagen I	3D	Randomly orientated, wide-spreading, proliferation ↑, OCN ↑, RUNX2 ↑, OSX ↑, ALP n.a., Col I n.a.	[122]
	Nanofibers	+	PCL	3D	ALP ↑, BMP-2 ↑, RUNX2 ↑, Col I ↑, β-catenin ↑, Smad3 ↑	[118]
	Isotropic fibers	−	PCL	3D	Proliferation ↑, ALP ↑, Alizarin Red ↑	[114]
Myogenic	Fibers aligned	+	PCL/PCU	3D	Proliferation ↑, myosin ↑, tropomyosin ↑	[123]
Endothelial	Grooves	+	Quartz	2D	Spindle-shaped morphology, proliferation ↑, PECAM-1 ↑, vWF ↑, VE-cadherin ↑, tube formation ↑	[119]
	Dual-scale sinusoidal grooves	+	Polystyrene	2D	Proliferation ↑, ac-LDL ↑	[120]
Neurogenic	Network pattern	+	Graphene oxid	2D	Spreading ↓, Tuj-1 ↑, length↑	[121]
	Grooves	+	PDA coated polystyrene	2D	Neurite length ↑, Tuj-1 ↑	[117]
Tendon	Nanofibers (663.5 nm)	+	PLGA	3D	Proliferation ↑, SCX ↑, TNC ↑, COL I ↑, TNMD ↑	[124]

#### 4.2.2. Tubes, Pores and Pillars

Table 3 gives an overview of studies investigating the effect of tubes, pores, and pillars on ASC behavior. Lv et al. investigate the effect of TiO_2_ nanotubes on the osteogenic differentiation of ASCs. They found that ASCs seeded on nanotubes and cultured without soluble osteogenic factors in a culture medium differentiated into the osteogenic direction. Furthermore, they found that the optimal diameter of nanotubes for osteogenic differentiation was 70 nm [125]. These results are in line with Ehlert et al. who showed osteogenic differentiation of ASCs cultured on pores/tubes with 15–65 nm in diameter without biochemical stimulation [126]. It can be hypothesized that TiO_2_ nanotubes and -pores in a diameter range from 70 nm to 110 nm replicate the porous structure of bone and creates better conditions for osteogenesis, whereas bigger pores provide enough space for the increase of the cell during adipogenesis. On the other hand, pore sizes that are too big fail to provide the necessary cell–cell and cell–matrix interactions for differentiation. Lemos et al. used pores with a diameter of 112 µm in Silk-ECM-carbon nanotube hydrogels [127]. For the ECM component, they used osteogenic cell-derived ECM from ASCs. As several studies demonstrated the inductive properties of natural ECM it can be hypothesized that in this study the ECM plays an important role in the induction of differentiation. Yang et al. investigated the chondrogenic differentiation of ASCs cultured in gelatin hydrogels with random or structured pores [128]. They found that in structured pores, ASCs are more likely to build cellular spheres and, therefore, cell–cell contacts enhancing chondrogenic differentiation are demonstrated by elevated GAG production, aggrecan, and SOX9 expression. It is known that chondrogenic differentiation of ASCs is best by culturing the cell as a pellet. In this form, the cells experience most in vivo-like conditions in terms of nutrient supply, cell-cell interaction, and space.

Not only the dimension of the structure but also the type of the structure seems to have an impact on cellular behavior. Park et al. investigated the adipogenic, chondrogenic, and osteogenic differentiation of ASCs cultured on flat TCPS materials or with 200 nm pores or pillars [102]. They found the highest chondrogenic on flat surfaces, the highest adipogenic differentiation on porous surfaces, and the highest osteogenic differentiation on pillars. Next to the classical differentiation makers they investigated integrin expression and found an upregulation of integrin α6 on pores and an upregulation of integrin α5 and α2 on pillars. Integrin α3 is downregulated on both structures. These results underline the importance of integrin expression and interaction in differentiation.

Several studies demonstrated osteogenic differentiation of ASCs cultured on pillar structures of various diameters (20 nm to 33.8 µm) and different materials. However, it has to be considered that in most studies only osteogenic differentiation was investigated. The expression of adipogenic or chondrogenic differentiation was not determined. Mukhopadhyay et al. showed an upregulation of differentiation genes for all three mesenchymal lineages in ASCs cultured on nodular structures without differentiation factors in the cell culture medium [129]. This study raises the question if there is an upregulation of differentiation markers for all mesenchymal lineages in ASCs cultured on specific topographies without chemical inducers in the cell culture medium. They further showed restrained senescence (downregulation of p53 and p21) and epithelial transition (upregulation of CDH1 and CK-19) in ASCs cultured on nodular structures. As substrate material, they used honey silk fibroin, which can be considered a highly bioactive material and might affect the impact of the topography. Similar results are generated by Wang et al. who investigated the expression of osteogenic, chondrogenic, and adipogenic genes in ASCs cultured on nodular structures with a diameter from 92 nm to 267 nm [130]. They found inhibition of spreading and proliferation. Regarding lineage-specific genes, BSP was upregulated whereas RUNX2 and OPN (osteogenic) and adipogenic genes PPARγ and adiponectin were not affected. Chondrogenic genes aggrecan, SOX9, and COL II were upregulated. Thus, the culture of ASCs on the nodular structures sees to induce an osteochondrogenic differentiation whereas adipogenic differentiation was not induced. An unusual structure was used by Ramaswamy et al. who used nature-inspired nodular structures as culture substrates. They blotted the surface of parsley, rose, and daisy leaves or petal in hydroxyapatite resulting in islet-like structures, honeycomb structures, and pillar structures [131]. Determination of the osteogenic differentiation of ASCs cultured on top of these surfaces revealed the highest RUNX2 expression and ALP activity on pillar structures.

**Table 3:** Overview of the influence of tubes/pores, pillars, and nodular structures on ASCs. (TiO_2_: titanium oxide, ECM: extracellular matrix, TCPS: tissue culture polystyrol, BCC: binary colloidal crystals).

Differentiation	Topography		Soluble Factors	Material		Results	Ref.
		15–65 nm	−	TiO_2_	2D	Proliferation ↑, ALP ↑	[126]
Osteogenic	Tubes/pores	70 nm	−/+	TiO_2_	2D	Adhesion ↑, proliferation ↑, ALP ↑, Alizarin Red ↑, RUNX2 ↑, OC ↑	[125]
		108 nm	+	TiO_2_	2D	Alizarin Red ↑, SP7 ↑, BGLAP ↑, RUNX2 n.a., SPARC n.a., BMP2 n.a., ALP n.a.	[132]
		112 µm	−	Silk-ECM-carbon nanotube hydrogels	2D	ALP ↑, RUNX2 ↑, OPN ↑, COL I ↑	[127]
Chondrogenic		100 µm	+	Gelatin hydrogel	3D	Proliferation ↑, GAG production ↑, aggrecan ↑, SOX9 ↑	[128]
Adipogenic		200 nm	+	TCPS	2D	Oil Red O ↑, FABP ↑, PPARγ ↑, GLUT4 ↑, integrin α6 ↑, integrin α3 ↓	[102]
Osteogenic	Pillars/nodules	20 nm	−	Silicon	2D	spreading ↓, Alizarin Red ↑, osteopontin ↑	[133]
		200 nm	+	TCPS	2D	Kossa staining ↑, BSP ↑, OCN ↑, RUNX2 ↑, integrin α5 ↑, integrin α2 ↑, integrin α3 ↓	[102]
		200 nm	−	Polyetherether-ketone	2D	Proliferation ↑, ALP ↑, RUNX2 ↑, OPN ↑, OCN ↑, Alizarin Red ↑,	[134]
		200–400 nm	−	Polystyrene	2D	200 nm + 500–750 nm: spreading ↓, viability ↓ 300 + 400 nm: spreading ↑, viability ↑ 200–400 nm: OCN ↑, OPN ↑, ALP ↑, RUNX2 ↑	[135]
		530 nm	−	Ti	2D	Alizarin Red ↑, ALP ↑	[136]
		33.8 µm	−	Hydroxyapatite	2D	Proliferation ↑, ALP ↑, RUNX2 ↑	[131]
		?	−	Hydroxyapatite	2D	Fibronectin absorbtion ↑, ALP ↑, BMP2 ↑, RUNX2 ↑, OCN ↑, OPN ↑, VEGF ↑	[137]
osteo-chondrogenic		92–267 nm	−	BCC	2D	Spreading ↓, proliferation ↓, BSP ↑, RUNX2 n.a., OPN n.a., AGG ↑, SOX9 ↑, COL II ↑, PPARγ n.a., adiponectin n.a.	[130]
adipogenic/chondrogenic/osteogenic		15 µm	−	Honey silk fibroin	2D	Spreading ↑, proliferation ↑, E-cadherin ↑, SOX9 ↑, RUNX2 ↑, PPARγ ↑, p53/p21 ↓, CDH1 ↑, CK-19 ↑	[129]

Regarding pore sizes, it can be assumed that for adipogenic differentiation it is important that the cell has enough space to adopt a rounded morphology and increase its size through lipid incorporation. Several studies demonstrated that chondrogenic differentiation is enhanced when the cells can accumulate. Thus, organized pores might be favorable to nodular or pillar topographies. Osteogenic differentiation seems to be enhanced by smaller pores and nodular or pillar structures, which might reflect the topographical features in native bone tissue.

#### 4.2.3. Cell-Imprints

Next to the man-made structures, several studies investigated the effect of cell-imprinted structures on different substrates (Table 4). For this technique instead of artificial structures, living cells serve as a template. Interestingly, for this type of surface topography, there is a quit unique picture regarding induction of differentiation: cell-imprinted surfaces induce the differentiation into the lineage that was the template without the supplementation of chemical factors into the medium. Bonakdar et al. produced cell-imprinted PDMS molds of dedifferentiated and mature chondrocytes and cultured ASCs in a growth medium on these molds [138]. They found that cells adopt the morphology of the particular cells used as a template. Further investigation of gene expression revealed an increase of chondrogenic genes (Col II, aggrecan, SOX9) in cells cultured on chondrocyte-imprinted structures. Furthermore, they demonstrated the redifferentiation of semifibroblasts cultured on chondrocyte-imprinted surfaces into chondrocytes and the transdifferentiation of tenocytes cultured on chondrocyte-imprinted substrates. Keyhanvar et al. and Mashinchian et al. obtained comparable results using keratinocytes [139,140]. Babaei et al. demonstrated this effect for osteoblast-imprinted PDMS [141]. In this study, the effect was further enhanced by chemical modification with bone lysate. Interestingly the highest differentiation was found for the combination of physical treatment (Ar plasma) and chemical modification with bone lysate compared to physical treatment or chemical modification alone.

These studies underline the importance of cell adhesion and morphology in the signaling pathway of differentiation. Solely, the guiding to a specific morphology leads to the differentiation of ASCs into a specific direction. One advantage of this technique is that these complex natural structures can be produced in every cell culture lab without expensive devices or materials. The greatest limitation of this method is that it is only 2D cell culture possible and there are limitations in the materials that can be used. The materials have to be castable at temperatures and pH that do not damage cells. Furthermore, the target cells need to be stable enough to serve as a template. Especially for mature adipocytes, this might become difficult. Also, the loadability of pre-adipocytes has to be determined, as the incorporated lipid vacuoles make the cell instable. Nazbar et al. demonstrated that also maintenance of stemness can be achieved by the culture of ASCs on PDMS imprints of undifferentiated ASCs [142]. Cells cultured on ASC-imprinted PDMS exhibited impaired adipogenic (Oil Red O and PPARγ) and osteogenic (Alizarin Red and OCN) differentiation compared to non-patterned PDMS. This approach is interesting for the long-term expansion and preservation of stem cell characteristics of ASCs or other stem cells concerning cell therapy.

**Table 4:** Overview of the influence of cell-imprinted structures on ASCs. (PDMS: polydimethylsiloxane.

Differentiation	Imprint	Soluble Factors	Material		Results	Ref.
Chondrogenic	Chondrocyte-imprint	−	PDMS	2D	Col I ↑, Col II ↑, aggrecan ↑, SOX9 ↑,	[138]
Keratinocytes	Keratinocyte-imprint	−	PDMS	2D	Cytokeratin 14 ↑, involucrin ↑, p63 ↑ keratin 10 ↑	[139,140]
Neurogenic	Neuronal-like cell-imprint	−	Chitosan/polyaniline	2D	Spreading ↑, GFAP ↑, MAP2 ↑	[143]
Osteogenic	Osteoblast-imprint	−	PDMS	2D	Proliferation ↑, ALP ↑, Alizarin Red ↑, RUNX2 ↑, OCN ↑, Col I ↓	[141]
Neurogenic	Schwann cell-imprint	−	PDMS	2D	P75 ↑, S100 ↑	[144]

The importance of cell morphology was underlined by studies investigating cell-imprints as culture substrates. There is a homogenous image of cell-imprints of differentiated cells that support differentiation into the respective lineage.

### 4.3. (Bio)Chemical Functionalization

Several studies demonstrated that on hydrophilic surfaces, adhesion and spreading of ASCs are enhanced [145,146,147]. A lot of used biomaterials, such as titanium or hydroxyapatite, are hydrophilic; however, many polymers used for tissue engineering are hydrophobic in their native state. Before cell seeding on these hydrophobic materials, surface modification is required to achieve sufficient cell adherence. One popular method to chemically functionalize surfaces is plasma treatment. Using different plasma results in functionalization with different chemical groups, such as NH_2_, COOH, CH_3_, or OH, which leads to hydrophilic surface properties. Kleinhans et al. demonstrated an altered MSC adhesion and focal adhesion assembly on polystyrol activated with different low-pressure plasma (NH_3_, CO_2_, AAc) [148]. They showed enhanced spreading and assembly of fibrillar adhesions on PS treated with NH_3_. Moreover, studies showed that different chemical and physical properties lead to the preferred adsorption of different proteins [149,150]. Depending on the composition of the cell culture medium and the surface characteristics, a distinct array of proteins is adsorbed on the surface that influences cellular adhesion by providing different attachment sites for the cells. In addition to the composition of adsorbed proteins, surface properties also influence the conformation of the adsorbed proteins affecting their biochemical functions. For example, Daum et al. demonstrated that hydrophilicity highly affects the conformational change of adsorbed fibronectin on oxygen plasma-treated PU surfaces. They found lower conformational change on more hydrophilic surfaces leading to higher adherence of primary endothelial cells [151]. Additionally, plasma treatment functionalization with ions or molecules is widely used to alter surface properties to enhance cellular attachment and regulate cellular behavior.

The simplest type of chemical surface modification is a modification with functional groups such as amine (-NH_2_), carboxyl (-COOH), and methyl (-CH_3_), using plasma technology (Table 5). For these modifications, there is surprising compliance for the results of investigating the influence on the differentiation potential of ASCs. Several studies found that amine-functionalization enhances osteogenic differentiation, whereas carboxyl-functionalization enhances chondrogenic differentiation and methyl-functionalization enhances adipogenic differentiation. Although in all studies soluble factors were used to induce differentiation, these corresponding results suggest an influence of the chemical functionalization on differentiation direction. This effect can be traced back to the different charges as a result of different functional groups and the subsequent impact on cell spreading. Amine functionalization leads to a positive and, therefore, hydrophilic surface that enhances the spreading of the cells. Carboxyl modification leads to spindle-like morphology and methyl modification leads to hydrophobic surfaces and subsequently rounded shape. As the morphology is closely linked to cell fate (spindle-like → osteogen/chondrogen; rounded → adipogenic), this explains the concordant results for the modification with functional groups.

Further, several studies demonstrated that also the modification of the surface with ions seems to be a promising tool in ASC differentiation (Table 5). Strontium is a widely used agent for stimulating osteogenesis. Aimaiti et al. demonstrated a dose-dependent effect of strontium in ASCs. Concentrations from 25 µM to 500 µM promoted osteogenic differentiation, whereas at concentrations from 1000 µM to 3000 µM, differentiation was inhibited and apoptosis was induced through ERK 1/2 phosphorylation accompanied by the downregulation of Bcl-2 and increased phosphorylation of BAX [152]. In line with the well-known osteoinductive effect of strontium, Kim et al. and Wei et al. found osteogenic differentiation of ASCs cultured on materials modified with strontium [153,154]. Next to the osteogenic markers, Kim et al. found the upregulation of Wnt and β-catenin. Furthermore, they found upregulation of integrin α2 and integrin β1 and 3. The upregulation of integrin α2 is in line with the findings of Park et al. who showed upregulation in ASCS cultured on nano pillar structures for osteogenic differentiation [102]. This effect in integrin α2 expression during osteogenesis was also found in other literature [155]. Bostancioglu et al. demonstrated that ASCs grown on modified hydroxyapatite, nano-coated with zinc/silver (Zn/Ag) or zinc/silver/copper (Zn/Ag/Cu), exhibit elevated ALP activity after 28 days of culture without supplements in the medium [156]. Focaroli et al. demonstrated chondrogenic differentiation using Ca/Co coated alginate beads without supplementation of chemical factors [157]. In this study, ASCs were encapsulated within the modified alginate beads providing a cartilage-like environment. The cells were not attached to a surface and can spread but have to keep a rounded shape within the hydrogel.

The classical way for chemical surface modification is coating the substrate or scaffold with synthetic or natural peptides or proteins to create in vivo-like circumstances. These include ECM proteins but also other peptides and polymers. Zhao et al. compared multilayers of collagen with hyaluronan (HA) or chondroitin sulfate (CS) [158]. They demonstrated enhanced proliferation, ALP activity, and expression of RUNX2 and Col I in ASC cultured on multilayers containing CS compared to HA. Thus, the type of GAG seems to have a great impact on cellular behavior. 

Polydopamine is a relatively new material exhibiting antibacterial properties via amine structures and hydrogen peroxide that denature the bacterial cell membrane [159,160]. Polydopamine has been used extensively in the modification of various materials to generate novel material properties. It can stick to almost any substrate including metals, polymers, and extra hydrophobic surfaces. The chemical structure is rich in functional groups that can be used for further modification [161]. Kao et al. and Lin et al. demonstrated the osteogenic differentiation of ASCs cultured on PLA fiber mats and scaffolds modified with polydopamine [162,163]. Additionally, they found a pro-angiogenic effect of the polydopamine coating. For laminin, there is evidence to enhance neuronal differentiation. Lee et al. compared the differentiation capacity of ASCs and BM MSCs (see also Table 1). Next to the impact of stiffness, they found enhanced neuronal differentiation of ASCs cultured on laminin coating. Foldberg et al. investigated the differentiation (neurogenic, myogenic, chondrogenic, osteogenic, adipogenic, and endothelial) of ASCs cultured on PLA-coated glass substrates [164]. They found an extensive upregulation of myogenic genes MyoD and Myf5. This effect was further enhanced by patterned substrates exhibiting pore-like topography with a diameter of 250 nm.

**Table 5:** Overview of the influence of (bio)chemical functionalization on ASCs. (HAP: hydroxyapatite, PCL: poly caprolactone, PLA: polylactide, CS: chondroitin sulfate).

Differentiation	Functionalization	Soluble Factors	Material		Results	Ref.
Osteogenic	NH_2_	+	polystyrene	2D	ALP ↑ spreading ↑, RUNX2 ↑ spreading ↑, focal adhesion ↑, ALP ↑, Col I ↑, OSC ↑	[165,166,167,168,169]
	Strontium	+/−	Ti HAP	2D 3D	Focal adhesion ↑, Integrin α2 ↑, Integrin β1/β3 ↑, RhoA ↑, proliferation ↓, ALP ↑, RUNX2 ↑, BSP ↑, OC ↑, Wnt ↑, β-catenin ↑ ALP ↑, BMP2 ↑, RUNX2 ↑, OCN ↑, OX ↑, VEGF ↑	[153,154]
	Zink/Silver and zink/Silver/Copper	−	HAP	2D	ALP ↑	[156]
	MgO	+	PCL	3D	Viability ↑, ALP ↑, calcium ↑, RUNX2 ↑, Col I ↑, OPN ↑	[170]
	Graphene	−	Ti	3D	ALP ↑, bone regeneration ↑	[171]
	Poly-dopamine	+	PLA	3D	Proliferation ↑, ALP ↑, OC ↑, Alizarin Red ↑, vWF ↑, Ang1 ↑	[162,163]
	CS (+Col I)	+	Glass	2D	Proliferation ↑, ALP ↑, RUNX2 ↑, Col I ↑	[158]
Chondrogenic	COOH	+	Polystyrene	2D	RUNX2 ↑, Collagen II ↑ spreading ↑, focal adhesion ↑, aggrecan ↑, Col II ↑	[165,169]
	Ca/Co alginate beads	−	Alginate	3D	Sox9 ↑, VCAN ↑, Coll II ↑	[157]
Adipogenic	CH_3_	+	Polystyrene	2D	Spreading ↓, migration ↑, PPARγ ↑,	[169]
Myogenic	PLA	−	Glass	2D	MyoD ↑, Myf5 ↑	[164]
Neurogenic	Laminin	−	Poly-acrylamide	2D	Β3-tubulin ↑	[92]

Surface functionalization with chemical groups affects its hydrophilicity and subsequent protein adsorption and integrin binding/signaling. Methyl groups were shown to provide a more adipogenic environment whereas amine groups were shown to enhance the osteogenic environment. Further, strontium ions are well known to support osteogenic differentiation. To generate a chondrogenic surrounding, functionalization with carboxyl groups was shown to play an important role.

#### Extracellular Matrix

Simplicity in generation, application, and processing are advantages of the use of single ECM proteins as a biomaterial. In vivo, all cells in all tissues are surrounded by different ECM proteins—the tissue-specific ECM. Therefore, it is implausible that one specific protein induces a specific direction of differentiation. Instead of one protein, it is more likely that a tissue-specific combination of different proteins triggers a specific cellular behavior. As the natural ECM is a complex network of various molecules, to date, it is not possible to rebuild the natural ECM. Thus, researchers focus on the use of natural ECM as a biomaterial. To date, this natural ECM can be generated in two ways: (i) decellularization of native tissues/organs (dECM) and (ii) generation of cell-derived ECM (cdECM).

As the ECM is the natural environment of the cells in tissues and provides a complex orchestrated set of physicochemical cues both—native dECM and cdECM—are popularly used biomaterials (Table 6). Natural ECM combines all types of tissue-specific characteristics: stiffness, topography, and chemical cues by ECM-bound growth factors and other signaling molecules [172]. Several studies demonstrated that tissue-specific ECM induces differentiation into the cell type matching the origin tissue or cell type of the ECM. Kim et al. investigated the use of decellularized muscle and tendon ECM for the bioprinting of the muscle–tendon junction zone [173]. They found upregulation of myogenic genes in ASCs cultured with muscle ECM and upregulation of tenogenic genes in ASCs cultured with tendon ECM without chemical differentiation factors. Moreover, the fabricated tissues exhibit well-aligned morphological structures. Tang et al. investigated the adipogenic capacity of decellularized adipose tissue compared to dermal tissue [174]. Interestingly, they found higher adipogenic differentiation using dermal ECM compared to adipose ECM in in vitro as well as in vivo experiments. In contrast, hydrogels decellularized ECM from various tissues were shown to induce and enhance the differentiation of ASCs into the respective lineage [175]. An important point in the use of ECM as a biomaterial is the processing of the ECM. Decellularization agents, such as SDS, are known to significantly affect ECM composition by destructing GAGs. Further, the use of different digestion enzymes (e.g., α-amylase, collagenase, and pepsin) for the preparation of ECM hydrogels has a great impact on ECM bioactivity [82]. Chemical modification of dECM allows the integration of additional functionalities [176]. However, chemical modification of native dECM alters the integrity of the natural structure of the ECM. In contrast, chemical modification of cdECM can be achieved by the incorporation of specifically addressable functional groups by metabolic glycoengineering [177,178]. This method allows the modification of cdECM with various molecules without affecting the chemical and structural integrity. For the effect of cdECM from different cell types on the ASCs’ behavior, there is great consensus. However, the induction of differentiation by chemical factors in the cell culture medium has a greater impact on ASCs than the ECM material.

## 5. Conclusions and Further Perspectives

Material properties are an important tool to influence ASCs’ behavior and fate. Table 7 gives an overview of the specific characteristics that can be identified to induce or enhance adipogenic, chondrogenic, or osteogenic differentiation. The most consistent results were found for material stiffness. As tissue stiffness changes during development, it would be interesting to investigate the influence of adjustable stiffness over the culture period.

However, the comparison of the different studies is complicated by using different materials, functionalization, and culture parameters, including a batch of FCS and culture time. Further results of the studies strongly depend on the determined outcomes. For example, gene expression is more sensitive compared to histological staining and is also able to detect differentiation in an earlier state. It seems that surface characteristics only enhance specific differentiation directions instead of inducing differentiation itself. Mostly, there are only individual studies investigating certain modifications, which makes it difficult and generalize the results. To make a clear statement, more extensive investigations should be made systematically examining the effect of individual characteristics and combinations of them. Moreover, these examinations should be made without induction of differentiation by soluble factors in the culture medium to exclude this influence. To achieve reliable results, the effect of material properties on differentiation into all of the three main differentiation directions (adipogenic, chondrogenic, and osteogenic) should be investigated.

**Table 7:** Overview of material characteristics that support adipogenic, chondrogenic, and osteogenic differentiation of ASCs.

Differentiation	Material Characteristics
Adipogenic	Softer materials (comparable to native tissue), lager pores that allow rounded shape and lipid storage, surface functionalization with methyl groups adipose tissue-derived and pre-adipocyte-derived ECM.
Chondrogenic	Material stiffness in the medium range, topography that allows the spheroid formation and chondrocyte imprint, surface functionalization with carboxy groups chondrocyte-derived ECM.
Osteogenic	Stiff materials, smaller pores, aligned fiber/grooves and nodular or pillar structures and osteoblast imprint, surface functionalization with amine groups or strontium bone tissue-derived and pre-osteoblast-derived ECM.

## Figures and Tables

**Table 6:** Overview of the influence of natural ECM from native tissue and cultured cells on ASCs.

	Tissue/Cell-Source	Soluble Factors	Material	Results	Ref.
Native ECM	Muscle tissue	−	Bioink	Myogenic: α-smooth muscle actin ↑, myosin ↑	[179]
		−	Coating/bioink	Myogenic: MyoD ↑, Myh2 ↑	[173]
	Adipose tissue	−	Hydrogel	Adipogenic: Proliferation ↑, Oil Red O ↑	[175]
		+	Hydrogel	Adipogenic: adiponectin ↑, FABP4 ↑, PPARγ ↑	[82,176,180,181]
	Liver tissue	+		Hepatocyte-like: AFP ↑, PCK ↑, CYP ↑, CK-18 ↑	[182,183]
	Dermis	−	Hydrogel	Adipogenic: PPARγ ↑	[174]
	Tendon	−	Coating/bioink	Tendon: Scx ↑, Tnmd ↑	[173]
	Bone	+	Hydrogel	Osteogenic: ALP ↑	[181]
Cell-derived ECM	Pre-adipocytes	−	Coating	Adipogenic: PPARγ ↑, adiponectin ↑	[184]
		+	Coating	Adipogenic: PPARγ ↑, FABP4 ↑	[185]
	Pre-chondrocytes	+	Coating	Chondrogenic: Col IX ↑	[186]
	Pre-osteoblasts	−	Coating	Osteogenic: Acan ↑, Col I ↑, ALP ↑, SPP1 ↑, Col IX ↑	[186]
	Adipose-derived stem cells	+	Coating	Adipogenic: PPARγ ↑, CEBPα ↑	[187]

## Data Availability

Not applicable.

## References

[1] Kshitiz, Park J., Kim P., Helen W., Engler A.J., Levchenko A., Kim D.H. (2012). Control of stem cell fate and function by engineering physical microenvironments. Integr. Biol..

[2] Han S.-B., Kim J.-K., Lee G., Kim D.-H., Han S.-B., Kim J.-K., Lee G.D., Kim D.-H. (2020). Mechanical Properties of Materials for Stem Cell Differentiation. Adv. Biosyst..

[3] Khan A.U., Qu R., Fan T., Ouyang J., Dai J. (2020). A glance on the role of actin in osteogenic and adipogenic differentiation of mesenchymal stem cells. Stem Cell Res. Ther..

[4] Gimble J.M., Katz A.J., Bunnell B.A. (2007). Adipose-Derived Stem Cells for Regenerative Medicine. Circ. Res..

[5] Zhu Y., Liu T., Song K., Fan X., Ma X., Cui Z. (2008). Adipose-derived stem cell: A better stem cell than BMSC. Cell Biochem. Funct..

[6] Jung S., Panchalingam K.M., Rosenberg L., Behie L.A. (2012). Ex vivo expansion of human mesenchymal stem cells in defined serum-free media. Stem Cells Int..

[7] Dominici M., Le Blanc K., Mueller I., Slaper-Cortenbach I., Marini F., Krause D.S., Deans R.J., Keating A., Prockop D.J., Horwitz E.M. (2006). Minimal criteria for defining multipotent mesenchymal stromal cells. The International Society for Cellular Therapy position statement. Cytotherapy.

[8] Trojahn Kølle S.F., Oliveri R.S., Glovinski P.V., Kirchhoff M., Mathiasen A.B., Elberg J.J., Andersen P.S., Drzewiecki K.T., Fischer-Nielsen A. (2013). Pooled human platelet lysate versus fetal bovine serum-investigating the proliferation rate, chromosome stability and angiogenic potential of human adipose tissue-derived stem cells intended for clinical use. Cytotherapy.

[9] Zuk P.A., Zhu M., Mizuno H., Huang J., Futrell J.W., Katz A.J., Benhaim P., Lorenz H.P., Hedrick M.H. (2001). Multilineage cells from human adipose tissue: Implications for cell-based therapies. Tissue Eng..

[10] Scioli M.G., Bielli A., Gentile P., Mazzaglia D., Cervelli V., Orlandi A. (2014). The biomolecular basis of adipogenic differentiation of adipose-derived stem cells. Int. J. Mol. Sci..

[11] Mildmay-White A., Khan W. (2017). Cell Surface Markers on Adipose-Derived Stem Cells: A Systematic Review. Curr. Stem Cell Res. Ther..

[12] Peng L., Jia Z., Yin X., Zhang X., Liu Y., Chen P., Ma K., Zhou C. (2008). Comparative Analysis of Mesenchymal Stem Cells from Bone Marrow, Cartilage, and Adipose Tissue. Stem Cells Dev..

[13] Pittenger M.F., Mackay A.M., Beck S.C., Jaiswal R.K., Douglas R., Mosca J.D., Moorman M.A., Simonetti D.W., Craig S., Marshak D.R. (1999). Multilineage potential of adult human mesenchymal stem cells. Science.

[14] Mazini L., Rochette L., Admou B., Amal S., Malka G. (2020). Hopes and Limits of Adipose-Derived Stem Cells (ADSCs) and Mesenchymal Stem Cells (MSCs) in Wound Healing. Int. J. Mol. Sci..

[15] Trzyna A., Banaś-Ząbczyk A. (2021). Adipose-Derived Stem Cells Secretome and Its Potential Application in “Stem Cell-Free Therapy”. Biomolecules.

[16] Kapur S.K., Katz A.J. (2013). Review of the adipose derived stem cell secretome. Biochimie.

[17] Li P., Guo X. (2018). A review: Therapeutic potential of adipose-derived stem cells in cutaneous wound healing and regeneration 11 Medical and Health Sciences 1103 Clinical Sciences 10 Technology 1004 Medical Biotechnology. Stem Cell Res. Ther..

[18] Frese L., Dijkman P.E., Hoerstrup S.P. (2016). Adipose Tissue-Derived Stem Cells in Regenerative Medicine. Transfus. Med. Hemotherapy.

[19] Alió Del Barrio J.L., El Zarif M., De Miguel M.P., Azaar A., Makdissy N., Harb W., El Achkar I., Arnalich-Montiel F., Alió J.L. (2017). Cellular Therapy with Human Autologous Adipose-Derived Adult Stem Cells for Advanced Keratoconus. Cornea.

[20] Jurado M., De La Mata C., Ruiz-García A., López-Fernández E., Espinosa O., Remigia M.J., Moratalla L., Goterris R., García-Martín P., Ruiz-Cabello F. (2017). Adipose tissue-derived mesenchymal stromal cells as part of therapy for chronic graft-versus-host disease: A phase I/II study. Cytotherapy.

[21] Pourmand G., Arjmand B., Safavi M., Heidari R., Aghayan H., Bazargani S.T., Dehghani S., Goodarzi P., Mohammadi-Jahani F., Heidari F. (2017). Concomitant Transurethral and Transvaginal-Periurethral Injection of Autologous Adipose Derived Stem Cells for Treatment of Female Stress Urinary Incontinence: A Phase One Clinical Trial. Acta Med. Iran..

[22] Sarveazad A., Newstead G.L., Mirzaei R., Joghataei M.T., Bakhtiari M., Babahajian A., Mahjoubi B. (2017). A new method for treating fecal incontinence by implanting stem cells derived from human adipose tissue: Preliminary findings of a randomized double-blind clinical trial. Stem Cell Res. Ther..

[23] Tsai Y.A., Liu R.S., Lirng J.F., Yang B.H., Chang C.H., Wang Y.C., Wu Y.S., Ho J.H.C., Lee O.K., Soong B.W. (2017). Treatment of Spinocerebellar Ataxia With Mesenchymal Stem Cells: A Phase I/IIa Clinical Study. Cell Transplant..

[24] Bailey A.M., Kapur S., Katz A.J. (2010). Characterization of Adipose-Derived Stem Cells: An Update. Curr. Stem Cell Res. Ther..

[25] Farmer S.R. (2005). Regulation of PPARγ activity during adipogenesis. Int. J. Obes..

[26] Bruderer M., Richards R.G., Alini M., Stoddart M.J. (2014). Role and regulation of RUNX2 in osteogenesis. Eur. Cells Mater..

[27] Yi S.W., Kim H.J., Oh H.J., Shin H., Lee J.S., Park J.S., Park K.H. (2018). Gene expression profiling of chondrogenic differentiation by dexamethasone-conjugated polyethyleneimine with SOX trio genes in stem cells. Stem Cell Res. Ther..

[28] Akiyama H., Chaboissier M.C., Martin J.F., Schedl A., De Crombrugghe B. (2002). The transcription factor Sox9 has essential roles in successive steps of the chondrocyte differentiation pathway and is required for expression of Sox5 and Sox6. Genes Dev..

[29] Nishimura R., Hata K., Ikeda F., Ichida F., Shimoyama A., Matsubara T., Wada M., Amano K., Yoneda T. (2008). Signal transduction and transcriptional regulation during mesenchymal cell differentiation. J. Bone Miner. Metab..

[30] Frith J., Genever P. (2008). Transcriptional control of mesenchymal stem cell differentiation. Transfus. Med. Hemother..

[31] Lefterova M.I., Zhang Y., Steger D.J., Schupp M., Schug J., Cristancho A., Feng D., Zhuo D., Stoeckert C.J., Liu X.S. (2008). PPARγ and C/EBP factors orchestrate adipocyte biology via adjacent binding on a genome-wide scale. Genes Dev..

[32] Nielsen R., Pedersen T.Å., Hagenbeek D., Moulos P., Siersbæk R., Megens E., Denissov S., Børgesen M., Francoijs K.J., Mandrup S. (2008). Genome-wide profiling of PPARγ:RXR and RNA polymerase II occupancy reveals temporal activation of distinct metabolic pathways and changes in RXR dimer composition during adipogenesis. Genes Dev..

[33] Rosen E.D., Sarraf P., Troy A.E., Bradwin G., Moore K., Milstone D.S., Spiegelman B.M., Mortensen R.M. (1999). PPARγ is required for the differentiation of adipose tissue in vivo and in vitro. Mol. Cell.

[34] Wu Z., Rosen E.D., Brun R., Hauser S., Adelmant G., Troy A.E., McKeon C., Darlington G.J., Spiegelman B.M. (1999). Cross-regulation of C/EBPα and PPARγ controls the transcriptional pathway of adipogenesis and insulin sensitivity. Mol. Cell.

[35] Li Y., Ge C., Long J.P., Begun D.L., Rodriguez J.A., Goldstein S.A., Franceschi R.T. (2012). Biomechanical Stimulation of Osteoblast Gene Expression Requires Phosphorylation of the RUNX2 Transcription Factor. J. Bone Miner. Res..

[36] Nakashima K., Zhou X., Kunkel G., Zhang Z., Deng J.M., Behringer R.R., De Crombrugghe B. (2002). The novel zinc finger-containing transcription factor Osterix is required for osteoblast differentiation and bone formation. Cell.

[37] Barczyk M., Carracedo S., Gullberg D. (2010). Integrins. Cell Tissue Res..

[38] Olivares-Navarrete R., Raz P., Zhao G., Chen J., Wieland M., Cochran D.L., Chaudhri R.A., Ornoy A., Boyan B.D., Schwartz Z. (2008). Integrin α2β1 plays a critical role in osteoblast response to micron-scale surface structure and surface energy of titanium substrates. Proc. Natl. Acad. Sci. USA.

[39] Gronthos S., Simmons P.J., Graves S.E., Robey P.G. (2001). Integrin-mediated interactions between human bone marrow stromal precursor cells and the extracellular matrix. Bone.

[40] Sun M., Chi G., Li P., Lv S., Xu J., Xu Z., Xia Y., Tan Y., Xu J., Li L. (2018). Effects of Matrix Stiffness on the Morphology, Adhesion, Proliferation and Osteogenic Differentiation of Mesenchymal Stem Cells. Int. J. Med. Sci..

[41] Liu J., DeYoung S.M., Zhang M., Zhang M., Cheng A., Saltiel A.R. (2005). Changes in integrin expression during adipocyte differentiation. Cell Metab..

[42] Uetaki M., Onishi N., Oki Y., Shimizu T., Sugihara E., Sampetrean O., Watanabe T., Yanagi H., Suda K., Fujii H. (2022). Regulatory roles of fibronectin and integrin α5 in reorganization of the actin cytoskeleton and completion of adipogenesis. Mol. Biol. Cell.

[43] Hamidouche Z., Fromigué O., Ringe J., Häupl T., Vaudin P., Pagès J.C., Srouji S., Livne E., Marie P.J. (2009). Priming integrin α5 promotes human mesenchymal stromal cell osteoblast differentiation and osteogenesis. Proc. Natl. Acad. Sci. USA.

[44] Wang H., Li J., Zhang X., Ning T., Ma D., Ge Y., Xu S., Hao Y., Wu B. (2018). Priming integrin alpha 5 promotes the osteogenic differentiation of human periodontal ligament stem cells due to cytoskeleton and cell cycle changes. J. Proteom..

[45] Changede R., Sheetz M. (2017). Integrin and cadherin clusters: A robust way to organize adhesions for cell mechanics. BioEssays.

[46] Parsons J.T., Horwitz A.R., Schwartz M.A. (2010). Cell adhesion: Integrating cytoskeletal dynamics and cellular tension. Nat. Rev. Mol. Cell Biol..

[47] Sneider A., Hah J., Wirtz D., Kim D.H. (2019). Recapitulation of molecular regulators of nuclear motion during cell migration. Cell Adhes. Migr..

[48] Cho S., Irianto J., Discher D.E. (2017). Mechanosensing by the nucleus: From pathways to scaling relationships. J. Cell Biol..

[49] McBeath R., Pirone D.M., Nelson C.M., Bhadriraju K., Chen C.S. (2004). Cell shape, cytoskeletal tension, and RhoA regulate stem cell lineage commitment. Dev. Cell.

[50] Yadav V., Sun L., Panilaitis B., Kaplan D.L. (2015). In vitro chondrogenesis with lysozyme susceptible bacterial cellulose as a scaffold. J. Tissue Eng. Regen. Med..

[51] Yao X., Peng R., Ding J. (2013). Effects of aspect ratios of stem cells on lineage commitments with and without induction media. Biomaterials.

[52] Wang X., Li S., Yan C., Liu P., Ding J. (2015). Fabrication of RGD micro/nanopattern and corresponding study of stem cell differentiation. Nano Lett..

[53] Clark E.A., Brugge J.S. (1995). Integrins and Signal Transduction Pathways: The Road Taken. Science.

[54] Aiyelabegan H.T., Sadroddiny E. (2017). Fundamentals of protein and cell interactions in biomaterials. Biomed. Pharmacother..

[55] Schwartz M.A. (2010). Integrins and extracellular matrix in mechanotransduction. Cold Spring Harb. Perspect. Biol..

[56] DeMali K.A., Sun X., Bui G.A. (2014). Force transmission at cell-cell and cell-matrix adhesions. Biochemistry.

[57] Prestwich T.C., MacDougald O.A. (2007). Wnt/beta-catenin signaling in adipogenesis and metabolism. Curr. Opin. Cell Biol..

[58] Christodoulides C., Lagathu C., Sethi J.K., Vidal-Puig A. (2009). Adipogenesis and WNT signalling. Trends Endocrinol. Metab..

[59] Pospisilik J.A., Schramek D., Schnidar H., Cronin S.J.F., Nehme N.T., Zhang X., Knauf C., Cani P.D., Aumayr K., Todoric J. (2010). Drosophila Genome-wide Obesity Screen Reveals Hedgehog as a Determinant of Brown versus White Adipose Cell Fate. Cell.

[60] Cousin W., Fontaine C., Dani C., Peraldi P. (2007). Hedgehog and adipogenesis: Fat and fiction. Biochimie.

[61] Chen L., Shi K., Frary C.E., Ditzel N., Hu H., Qiu W., Kassem M. (2015). Inhibiting actin depolymerization enhances osteoblast differentiation and bone formation in human stromal stem cells. Stem Cell Res..

[62] Xie J., Zhang D., Zhou C., Yuan Q., Ye L., Zhou X. (2018). Substrate elasticity regulates adipose-derived stromal cell differentiation towards osteogenesis and adipogenesis through β-catenin transduction. Acta Biomater..

[63] Johnson M.L., Rajamannan N. (2006). Diseases of Wnt signaling. Rev. Endocr. Metab. Disord..

[64] Day T.F., Guo X., Garrett-Beal L., Yang Y. (2005). Wnt/beta-catenin signaling in mesenchymal progenitors controls osteoblast and chondrocyte differentiation during vertebrate skeletogenesis. Dev. Cell.

[65] Takada I., Kouzmenko A.P., Kato S. (2009). Wnt and PPARγ signaling in osteoblastogenesis and adipogenesis. Nat. Rev. Rheumatol..

[66] Lecarpentier Y., Claes V., Duthoit G., Hébert J.L. (2014). Circadian rhythms, Wnt/beta-catenin pathway and PPAR alpha/gamma profiles in diseases with primary or secondary cardiac dysfunction. Front. Physiol..

[67] Chung S.S., Lee J.S., Kim M., Ahn B.Y., Jung H.S., Lee H.M., Kim J.W., Park K.S. (2012). Regulation of Wnt/beta-catenin signaling by CCAAT/enhancer binding protein β during adipogenesis. Obesity.

[68] Krishnan V., Bryant H.U., MacDougald O.A. (2006). Regulation of bone mass by Wnt signaling. J. Clin. Investig..

[69] Boland G.M., Perkins G., Hall D.J., Tuan R.S. (2004). Wnt 3a promotes proliferation and suppresses osteogenic differentiation of adult human mesenchymal stem cells. J. Cell. Biochem..

[70] Salzig D., Leber J., Merkewitz K., Lange M.C., Köster N., Czermak P. (2016). Attachment, Growth, and Detachment of Human Mesenchymal Stem Cells in a Chemically Defined Medium. Stem Cells Int..

[71] Berrier A.L., Yamada K.M. (2007). Cell–matrix adhesion. J. Cell. Physiol..

[72] Hong J.H., Hwang E.S., McManus M.T., Amsterdam A., Tian Y., Kalmukova R., Mueller E., Benjamin T., Spiegelman B.M., Sharp P.A. (2005). TAZ, a transcriptional modulator of mesenchymal stem cell differentiation. Science.

[73] Kirby T.J., Lammerding J. (2018). Emerging views of the nucleus as a cellular mechanosensor. Nat. Cell Biol..

[74] Mathieu P.S., Loboa E.G. (2012). Cytoskeletal and Focal Adhesion Influences on Mesenchymal Stem Cell Shape, Mechanical Properties, and Differentiation Down Osteogenic, Adipogenic, and Chondrogenic Pathways. Tissue Eng. Part B Rev..

[75] Huang G., Wang L., Wang S., Han Y., Wu J., Zhang Q., Xu F., Lu T.J. (2012). Engineering three-dimensional cell mechanical microenvironment with hydrogels. Biofabrication.

[76] Guimarães C.F., Gasperini L., Marques A.P., Reis R.L. (2020). The stiffness of living tissues and its implications for tissue engineering. Nat. Rev. Mater..

[77] Stevens L.R., Gilmore K.J., Wallace G.G., in het Panhuis M. (2016). Tissue engineering with gellan gum. Biomater. Sci..

[78] Pacelli S., Paolicelli P., Petralito S., Subham S., Gilmore D., Varani G., Yang G., Lin D., Casadei M.A., Paul A. (2020). Investigating the Role of Polydopamine to Modulate Stem Cell Adhesion and Proliferation on Gellan Gum-Based Hydrogels. ACS Appl. Bio Mater..

[79] Albrecht F.B., Dolderer V., Nellinger S., Schmidt F.F., Kluger P.J. (2022). Gellan Gum Is a Suitable Biomaterial for Manual and Bioprinted Setup of Long-Term Stable, Functional 3D-Adipose Tissue Models. Gels.

[80] Kumar S., Weaver V.M. (2009). Mechanics, malignancy, and metastasis: The force journey of a tumor cell. Cancer Metastasis Rev..

[81] Major L.G., Choi Y.S. (2018). Developing a high-throughput platform to direct adipogenic and osteogenic differentiation in adipose-derived stem cells. J. Tissue Eng. Regen. Med..

[82] Shridhar A., Lam A.Y.L., Sun Y., Simmons C.A., Gillies E.R., Flynn L.E. (2020). Culture on Tissue-Specific Coatings Derived from α-Amylase-Digested Decellularized Adipose Tissue Enhances the Proliferation and Adipogenic Differentiation of Human Adipose-Derived Stromal Cells. Biotechnol. J..

[83] Teong B., Wu S.C., Chang C.M., Chen J.W., Chen H.T., Chen C.H., Chang J.K., Ho M.L. (2018). The stiffness of a crosslinked hyaluronan hydrogel affects its chondro-induction activity on hADSCs. J. Biomed. Mater. Res. Part B Appl. Biomater..

[84] Zigon-Branc S., Markovic M., Van Hoorick J., Van Vlierberghe S., Dubruel P., Zerobin E., Baudis S., Ovsianikov A. (2019). Impact of Hydrogel Stiffness on Differentiation of Human Adipose-Derived Stem Cell Microspheroids. Tissue Eng. Part A.

[85] Sarangthem V., Singh T.D., Dinda A.K. (2021). Emerging Role of Elastin-Like Polypeptides in Regenerative Medicine. Adv. Wound Care.

[86] Gurumurthy B., Bierdeman P.C., Janorkar A.V. (2016). Composition of elastin like polypeptide-collagen composite scaffold influences in vitro osteogenic activity of human adipose derived stem cells. Dent. Mater..

[87] Newman K., Clark K., Gurumurthy B., Pal P., Janorkar A.V. (2020). Elastin-Collagen Based Hydrogels as Model Scaffolds to Induce Three-Dimensional Adipocyte Culture from Adipose Derived Stem Cells. Bioeng..

[88] Betre H., Ong S.R., Guilak F., Chilkoti A., Fermor B., Setton L.A. (2006). Chondrocytic differentiation of human adipose-derived adult stem cells in elastin-like polypeptide. Biomaterials.

[89] Kim C., Young J.L., Holle A.W., Jeong K., Major L.G., Jeong J.H., Aman Z.M., Han D.W., Hwang Y., Spatz J.P. (2020). Stem Cell Mechanosensation on Gelatin Methacryloyl (GelMA) Stiffness Gradient Hydrogels. Ann. Biomed. Eng..

[90] Banks J.M., Harley B.A.C., Bailey R.C. (2015). Tunable, Photoreactive Hydrogel System to Probe Synergies between Mechanical and Biomolecular Cues on Adipose-Derived Mesenchymal Stem Cell Differentiation. ACS Biomater. Sci. Eng..

[91] Guneta V., Loh Q.L., Choong C. (2016). Cell-secreted extracellular matrix formation and differentiation of adipose-derived stem cells in 3D alginate scaffolds with tunable properties. J. Biomed. Mater. Res. Part A.

[92] Lee J., Abdeen A.A., Tang X., Saif T.A., Kilian K.A. (2016). Matrix directed adipogenesis and neurogenesis of mesenchymal stem cells derived from adipose tissue and bone marrow. Acta Biomater..

[93] Zhang T., Lin S., Shao X., Shi S., Zhang Q., Xue C., Lin Y., Zhu B., Cai X. (2018). Regulating osteogenesis and adipogenesis in adipose-derived stem cells by controlling underlying substrate stiffness. J. Cell. Physiol..

[94] Allen J.L., Cooke M.E., Alliston T. (2012). ECM stiffness primes the TGFβ pathway to promote chondrocyte differentiation. Mol. Biol. Cell.

[95] Gao L., McBeath R., Chen C.S. (2010). Stem Cell Shape Regulates a Chondrogenic versus Myogenic Fate through Rac1 and N-cadherin. Stem Cells.

[96] Leight J.L., Wozniak M.A., Chen S., Lynch M.L., Chen C.S. (2012). Matrix rigidity regulates a switch between TGF-β1-induced apoptosis and epithelial-mesenchymal transition. Mol. Biol. Cell.

[97] Park J.S., Chu J.S., Tsou A.D., Diop R., Tang Z., Wang A., Li S. (2011). The effect of matrix stiffness on the differentiation of mesenchymal stem cells in response to TGF-β. Biomaterials.

[98] Young D.A., Choi Y.S., Engler A.J., Christman K.L. (2013). Stimulation of adipogenesis of adult adipose-derived stem cells using substrates that mimic the stiffness of adipose tissue. Biomaterials.

[99] Khoramgah M.S., Ranjbari J., Abbaszadeh H.A., Mirakabad F.S.T., Hatami S., Hosseinzadeh S., Ghanbarian H. (2020). Freeze-dried multiscale porous nanofibrous three dimensional scaffolds for bone regenerations. BioImpacts.

[100] Sun X., Tung W., Wang W., Xu X., Zou J., Gould O.E.C., Kratz K., Ma N., Lendlein A. (2019). The effect of stiffness variation of electrospun fiber meshes of multiblock copolymers on the osteogenic differentiation of human mesenchymal stem cells. Clin. Hemorheol. Microcirc..

[101] Ahn H.H., Lee I.W., Lee H.B., Kim M.S. (2014). Cellular Behavior of Human Adipose-Derived Stem Cells on Wettable Gradient Polyethylene Surfaces. Int. J. Mol. Sci..

[102] Park K.S., Cha K.J., Han I.B., Shin D.A., Cho D.W., Lee S.H., Kim D.S. (2012). Mass-producible Nano-featured Polystyrene Surfaces for Regulating the Differentiation of Human Adipose-derived Stem Cells. Macromol. Biosci..

[103] Tan A.W., Tay L., Chua K.H., Ahmad R., Akbar S.A., Pingguan-Murphy B. (2014). Proliferation and stemness preservation of human adipose-derived stem cells by surface-modified in situ TiO_2_ nanofibrous surfaces. Int. J. Nanomed..

[104] Yun Y.S., Kang E.H., Ji S., Lee S.B., Kim Y.O., Yun I.S., Yeo J.S. (2020). Quantitative Correlation of Nanotopography with Cell Spreading via Focal Adhesions Using Adipose-Derived Stem Cells. Adv. Biosyst..

[105] Yim E.K.F., Darling E.M., Kulangara K., Guilak F., Leong K.W. (2010). Nanotopography-induced changes in focal adhesions, cytoskeletal organization, and mechanical properties of human mesenchymal stem cells. Biomaterials.

[106] Mobasseri A., Faroni A., Minogue B.M., Downes S., Terenghi G., Reid A.J. (2015). Polymer Scaffolds with Preferential Parallel Grooves Enhance Nerve Regeneration. Tissue Eng. Part A.

[107] Howe A., Aplin A.E., Alahari S.K., Juliano R. (1998). Integrin signaling and cell growth control. Curr. Opin. Cell Biol..

[108] Chrzanowska-Wodnicka M., Burridge K. (1996). Rho-stimulated contractility drives the formation of stress fibers and focal adhesions. J. Cell Biol..

[109] Nobes C.D., Hall A. (1995). Rho, rac, and cdc42 GTPases regulate the assembly of multimolecular focal complexes associated with actin stress fibers, lamellipodia, and filopodia. Cell.

[110] Khatiwala C.B., Kim P.D., Peyton S.R., Putnam A.J. (2009). ECM Compliance Regulates Osteogenesis by Influencing MAPK Signaling Downstream of RhoA and ROCK. J. Bone Miner. Res..

[111] Jaiswal R.K., Jaiswal N., Bruder S.P., Mbalaviele G., Marshak D.R., Pittenger M.F. (2000). Adult Human Mesenchymal Stem Cell Differentiation to the Osteogenic or Adipogenic Lineage Is Regulated by Mitogen-activated Protein Kinase. J. Biol. Chem..

[112] Klees R.F., Salasznyk R.M., Kingsley K., Williams W.A., Boskey A., Plopper G.E. (2005). Laminin-5 induces osteogenic gene expression in human mesenchymal stem cells through an ERK-dependent pathway. Mol. Biol. Cell.

[113] Salasznyk R.M., Klees R.F., Hughlock M.K., Plopper G.E. (2009). ERK Signaling Pathways Regulate the Osteogenic Differentiation of Human Mesenchymal Stem Cells on Collagen I and Vitronectin. Cell Commun. Adhes..

[114] Calejo I., Reis R.L., Domingues R.M.A., Gomes M.E. (2022). Texturing Hierarchical Tissues by Gradient Assembling of Microengineered Platelet-Lysates Activated Fibers. Adv. Healthc. Mater..

[115] Calejo I., Costa-Almeida R., Reis R.L., Gomes M.E. (2019). A Textile Platform Using Continuous Aligned and Textured Composite Microfibers to Engineer Tendon-to-Bone Interface Gradient Scaffolds. Adv. Healthc. Mater..

[116] Ko E., Alberti K., Lee J.S., Yang K., Jin Y., Shin J., Yang H.S., Xu Q., Cho S.W. (2016). Nanostructured tendon-derived scaffolds for enhanced bone regeneration by human adipose-derived stem cells. ACS Appl. Mater. Interfaces.

[117] Chen C.H., Tsai C.C., Wu P.T., Wang I.K., Yu J., Tsai W.B. (2019). Modulation of Neural Differentiation through Submicron-Grooved Topography Surface with Modified Polydopamine. ACS Appl. Bio Mater..

[118] Xue R., Qian Y., Li L., Yao G., Yang L., Sun Y. (2017). Polycaprolactone nanofiber scaffold enhances the osteogenic differentiation potency of various human tissue-derived mesenchymal stem cells. Stem Cell Res. Ther..

[119] Shi Z., Neoh K.G., Kang E.T., Poh C.K., Wang W. (2014). Enhanced endothelial differentiation of adipose-derived stem cells by substrate nanotopography. J. Tissue Eng. Regen. Med..

[120] Kim H.W., Lee J.S., Park S.J., Rhie J.W., Kim D.S. (2020). Micro/Nano Dual-Scale Crossed Sinusoidal Wavy Patterns for Synergistic Promotion of Proliferation and Endothelial Differentiation of Human Adipose-Derived Stem Cells. Adv. Mater. Interfaces.

[121] Kim T.H., Shah S., Yang L., Yin P.T., Hossain M.K., Conley B., Choi J.W., Lee K.B. (2015). Controlling differentiation of adipose-derived stem cells using combinatorial graphene hybrid-pattern arrays. ACS Nano.

[122] Chen H., Qian Y., Xia Y., Chen G., Dai Y., Li N., Zhang F., Gu N. (2016). Enhanced Osteogenesis of ADSCs by the Synergistic Effect of Aligned Fibers Containing Collagen I. ACS Appl. Mater. Interfaces.

[123] Bayati V., Altomare L., Tanzi M.C., Farè S. (2013). Adipose-derived stem cells could sense the nano-scale cues as myogenic-differentiating factors. J. Mater. Sci. Mater. Med..

[124] Wu S., Zhou R., Zhou F., Streubel P.N., Chen S., Duan B. (2020). Electrospun thymosin β-4 loaded PLGA/PLA nanofiber/microfiber hybrid yarns for tendon tissue engineering application. Mater. Sci. Eng. C.

[125] Lv L., Liu Y., Zhang P., Zhang X., Liu J., Chen T., Su P., Li H., Zhou Y. (2015). The nanoscale geometry of TiO_2_ nanotubes influences the osteogenic differentiation of human adipose-derived stem cells by modulating H3K4 trimethylation. Biomaterials.

[126] Ehlert M., Radtke A., Jedrzejewski T., Roszek K., Bartmanski M., Piszczek P. (2020). In vitro studies on nanoporous, nanotubular and nanosponge-like titania coatings, with the use of adipose-derived stem cells. Materials.

[127] Lemos R., Maia F.R., Ribeiro V.P., Costa J.B., Coutinho P.J.G., Reis R.L., Oliveira J.M. (2021). Carbon nanotube-reinforced cell-derived matrix-silk fibroin hierarchical scaffolds for bone tissue engineering applications. J. Mater. Chem. B.

[128] Yang K.C., Chen I.H., Yang Y.T., Hsiao J.K., Wang C.C. (2020). Effects of scaffold geometry on chondrogenic differentiation of adipose-derived stem cells. Mater. Sci. Eng. C.

[129] Mukhopadhyay A., Das A., Mukherjee S., Rajput M., Gope A., Chaudhary A., Choudhury K., Barui A., Chatterjee J., Mukherjee R. (2021). Improved Mesenchymal Stem Cell Proliferation, Differentiation, Epithelial Transition, and Restrained Senescence on Hierarchically Patterned Porous Honey Silk Fibroin Scaffolds. ACS Appl. Bio Mater..

[130] Wang P.Y., Thissen H., Kingshott P. (2016). Stimulation of Early Osteochondral Differentiation of Human Mesenchymal Stem Cells Using Binary Colloidal Crystals (BCCs). ACS Appl. Mater. Interfaces.

[131] Ramaswamy Y., Roohani I., No Y.J., Madafiglio G., Chang F., Zhang F., Lu Z., Zreiqat H. (2021). Nature-inspired topographies on hydroxyapatite surfaces regulate stem cells behaviour. Bioact. Mater..

[132] Malec K., Góralska J., Hubalewska-Mazgaj M., Głowacz P., Jarosz M., Brzewski P., Sulka G.D., Jaskuła M., Wybrańska I. (2016). Effects of nanoporous anodic titanium oxide on human adipose derived stem cells. Int. J. Nanomed..

[133] Brammer K.S., Choi C., Frandsen C.J., Oh S., Jin S. (2011). Hydrophobic nanopillars initiate mesenchymal stem cell aggregation and osteo-differentiation. Acta Biomater..

[134] Zhang S., Feng Z., Hu Y., Zhao D., Guo X., Du F., Wang N., Sun C., Liu C., Liu H. (2022). Endowing Polyetheretherketone Implants with Osseointegration Properties: In Situ Construction of Patterned Nanorod Arrays. Small.

[135] Zhao C., Song X., Lu X. (2020). Directional osteo-differentiation effect of hadscs on nanotopographical self-assembled polystyrene nanopit surfaces. Int. J. Nanomed..

[136] Martel-Frachet V., Ivanova E.P., Le Clainche T., Linklater D., Wong S., Le P., Juodkazis S., Le Guevel X., Coll J.L. (2020). Mechano-bactericidal titanium surfaces for bone tissue engineering. ACS Appl. Mater. Interfaces.

[137] Wei Y., Liu L., Gao H., Shi X., Wang Y. (2020). In Situ Formation of Hexagon-like Column Array Hydroxyapatite on 3D-Plotted Hydroxyapatite Scaffolds by Hydrothermal Method and Its Effect on Osteogenic Differentiation. ACS Appl. Bio Mater..

[138] Bonakdar S., Mahmoudi M., Montazeri L., Taghipoor M., Bertsch A., Shokrgozar M.A., Sharifi S., Majidi M., Mashinchian O., Sekachaei M.H. (2016). Cell-Imprinted Substrates Modulate Differentiation, Redifferentiation, and Transdifferentiation. ACS Appl. Mater. Interfaces.

[139] Keyhanvar N., Zarghami N., Seifalian A., Keyhanvar P., Sarvari R., Salehi R., Rahbarghazi R., Ranjkesh M., Akbarzadeh M., Mahdipour M. (2022). The Combined Thermoresponsive Cell-Imprinted Substrate, Induced Differentiation, and “KLC Sheet” Formation. Adv. Pharm. Bull..

[140] Mashinchian O., Bonakdar S., Taghinejad H., Satarifard V., Heidari M., Majidi M., Sharifi S., Peirovi A., Saffar S., Taghinejad M. (2014). Cell-imprinted substrates act as an artificial niche for skin regeneration. ACS Appl. Mater. Interfaces.

[141] Babaei M., Nasernejad B., Sharifikolouei E., Shokrgozar M.A., Bonakdar S. (2022). Bioactivation of 3D Cell-Imprinted Polydimethylsiloxane Surfaces by Bone Protein Nanocoating for Bone Tissue Engineering. ACS Omega.

[142] Nazbar A., Samani S., Yazdian Kashani S., Amanzadeh A., Shoeibi S., Bonakdar S. (2022). Molecular imprinting as a simple way for the long-term maintenance of the stemness and proliferation potential of adipose-derived stem cells: An in vitro study. J. Mater. Chem. B.

[143] Eftekhari B.S., Eskandari M., Janmey P.A., Samadikuchaksaraei A., Gholipourmalekabadi M. (2021). Conductive chitosan/polyaniline hydrogel with cell-imprinted topography as a potential substrate for neural priming of adipose derived stem cells. RSC Adv..

[144] Dadashkhan S., Irani S., Bonakdar S., Ghalandari B. (2021). P75 and S100 gene expression induced by cell-imprinted substrate and beta-carotene to nerve tissue engineering. J. Appl. Polym. Sci..

[145] Webb K., Hlady V., Tresco P.A. (2000). Relationships among cell attachment, spreading, cytoskeletal organization, and migration rate for anchorage-dependent cells on model surfaces. J. Biomed. Mater. Res..

[146] Dalton B.A., Mc Farland C.D., Gengenbach T.R., Griesser H.J., Steele J.G. (2012). Polymer surface chemistry and bone cell migration. J. Biomater. Sci. Polym. Ed..

[147] Yang J., Shi G., Bei J., Wang S., Cao Y., Shang Q., Yang G., Wang W. (2002). Fabrication and surface modification of macroporous poly(L-lactic acid) and poly(L-lactic-co-glycolic acid) (70/30) cell scaffolds for human skin fibroblast cell culture. J. Biomed. Mater. Res..

[148] Kleinhans C., Schmohl L., Barz J., Kluger P.J. (2020). Low-pressure plasma activation enables enhanced adipose-derived stem cell adhesion. J. Biomed. Mater. Res. Part B Appl. Biomater..

[149] Firkowska-Boden I., Zhang X., Jandt K.D. (2018). Controlling Protein Adsorption through Nanostructured Polymeric Surfaces. Adv. Healthc. Mater..

[150] Wilson C.J., Clegg R.E., Leavesley D.I., Pearcy M.J. (2005). Mediation of biomaterial-cell interactions by adsorbed proteins: A review. Tissue Eng..

[151] Daum R., Mrsic I., Hutterer J., Junginger A., Hinderer S., Meixner A.J., Gauglitz G., Chassé T., Schenke-Layland K. (2021). Fibronectin adsorption on oxygen plasma-treated polyurethane surfaces modulates endothelial cell response. J. Mater. Chem. B.

[152] Aimaiti A., Maimaitiyiming A., Boyong X., Aji K., Li C., Cui L. (2017). Low-dose strontium stimulates osteogenesis but high-dose doses cause apoptosis in human adipose-derived stem cells via regulation of the ERK1/2 signaling pathway. Stem Cell Res. Ther..

[153] Kim H.S., Kim Y.J., Jang J.H., Park J.W. (2016). Surface Engineering of Nanostructured Titanium Implants with Bioactive Ions. J. Dent. Res..

[154] Wei Y., Gao H., Hao L., Shi X., Wang Y. (2020). Constructing a Sr^2+^-substituted surface hydroxyapatite hexagon-like microarray on 3d-plotted hydroxyapatite scaffold to regulate osteogenic differentiation. Nanomaterials.

[155] El-Rashidy A.A., El Moshy S., Radwan I.A., Rady D., Abbass M.M.S., Dörfer C.E., El-Sayed K.M.F. (2021). Effect of Polymeric Matrix Stiffness on Osteogenic Differentiation of Mesenchymal Stem/Progenitor Cells: Concise Review. Polymers.

[156] Bostancioglu R.B., Gurbuz M., Akyurekli A.G., Dogan A., Koparal A.S., Koparal A.T. (2017). Adhesion profile and differentiation capacity of human adipose tissue derived mesenchymal stem cells grown on metal ion (Zn, Ag and Cu) doped hydroxyapatite nano-coated surfaces. Colloids Surf. B Biointerfaces.

[157] Focaroli S., Teti G., Salvatore V., Orienti I., Falconi M. (2016). Calcium/Cobalt Alginate Beads as Functional Scaffolds for Cartilage Tissue Engineering. Stem Cells Int..

[158] Zhao M., Altankov G., Grabiec U., Bennett M., Salmeron-Sanchez M., Dehghani F., Groth T. (2016). Molecular composition of GAG-collagen I multilayers affects remodeling of terminal layers and osteogenic differentiation of adipose-derived stem cells. Acta Biomater..

[159] Xu Z., Wang T., Liu J. (2022). Recent Development of Polydopamine Anti-Bacterial Nanomaterials. Int. J. Mol. Sci..

[160] Fu Y., Yang L., Zhang J., Hu J., Duan G., Liu X., Li Y., Gu Z. (2021). Polydopamine antibacterial materials. Mater. Horiz..

[161] Li Y., Li C., Yu R., Ding Y. (2022). Application of polydopamine on the implant surface modification. Polym. Bull..

[162] Kao C.T., Lin C.C., Chen Y.W., Yeh C.H., Fang H.Y., Shie M.Y. (2015). Poly(dopamine) coating of 3D printed poly(lactic acid) scaffolds for bone tissue engineering. Mater. Sci. Eng. C.

[163] Lin C.C., Fu S.J. (2016). Osteogenesis of human adipose-derived stem cells on poly(dopamine)-coated electrospun poly(lactic acid) fiber mats. Mater. Sci. Eng. C.

[164] Foldberg S., Petersen M., Fojan P., Gurevich L., Fink T., Pennisi C.P., Zachar V. (2012). Patterned poly(lactic acid) films support growth and spontaneous multilineage gene expression of adipose-derived stem cells. Colloids Surf. B Biointerfaces.

[165] Griffin M.F., Ibrahim A., Seifalian A.M., Butler P.E.M., Kalaskar D.M., Ferretti P. (2017). Chemical group-dependent plasma polymerisation preferentially directs adipose stem cell differentiation towards osteogenic or chondrogenic lineages. Acta Biomater..

[166] Liu X., Feng Q., Bachhuka A., Vasilev K. (2014). Surface modification by allylamine plasma polymerization promotes osteogenic differentiation of human adipose-derived stem cells. ACS Appl. Mater. Interfaces.

[167] Chaves C., Alshomer F., Palgrave R.G., Kalaskar D.M. (2016). Plasma Surface Modification of Polyhedral Oligomeric Silsequioxane-Poly(carbonate-urea) Urethane with Allylamine Enhances the Response and Osteogenic Differentiation of Adipose-Derived Stem Cells. ACS Appl. Mater. Interfaces.

[168] Liu X., Shi S., Feng Q., Bachhuka A., He W., Huang Q., Zhang R., Yang X., Vasilev K. (2015). Surface Chemical Gradient Affects the Differentiation of Human Adipose-Derived Stem Cells via ERK1/2 Signaling Pathway. ACS Appl. Mater. Interfaces.

[169] Chieh H.F., Su F.C., Lin S.C., Shen M.R., Liao J. (2013). Der Migration patterns and cell functions of adipose-derived stromal cells on self-assembled monolayers with different functional groups. J. Biomater. Sci. Polym. Ed..

[170] Niknam Z., Golchin A., Rezaei-Tavirani M., Ranjbarvan P., Zali H., Omidi M., Mansouri V. (2022). Osteogenic Differentiation Potential of Adipose-Derived Mesenchymal Stem Cells Cultured on Magnesium Oxide/Polycaprolactone Nanofibrous Scaffolds for Improving Bone Tissue Reconstruction. Adv. Pharm. Bull..

[171] Sun X., Tong S., Yang S., Guo S. (2021). The effects of graphene on the biocompatibility of a 3D-printed porous Titanium alloy. Coatings.

[172] Nellinger S., Schmidt I., Heine S., Volz A.C., Kluger P.J. (2020). Adipose stem cell-derived extracellular matrix represents a promising biomaterial by inducing spontaneous formation of prevascular-like structures by mvECs. Biotechnol. Bioeng..

[173] Kim W.J., Kim G.H. (2022). A bioprinted complex tissue model for myotendinous junction with biochemical and biophysical cues. Bioeng. Transl. Med..

[174] Tang W., Qi J., Wang Q., Qu Y., Fu S., Luan J. (2022). Investigating the Adipogenic Effects of Different Tissue-Derived Decellularized Matrices. Front. Bioeng. Biotechnol..

[175] Zhao Y., Fan J., Bai S. (2019). Biocompatibility of injectable hydrogel from decellularized human adipose tissue in vitro and in vivo. J. Biomed. Mater. Res. Part B Appl. Biomater..

[176] Li S., Liu Y., McCann J., Ravnic D.J., Gimble J.M., Hayes D.J. (2022). Hybrid adipose graft materials synthesized from chemically modified adipose extracellular matrix. J. Biomed. Mater. Res. Part A.

[177] Ruff S.M., Keller S., Wieland D.E., Wittmann V., Tovar G.E.M., Bach M., Kluger P.J. (2017). clickECM: Development of a cell-derived extracellular matrix with azide functionalities. Acta Biomater..

[178] Nellinger S., Rapp M.A., Southan A., Wittmann V., Kluger P.J. (2022). An Advanced “clickECM” That Can be Modified by the Inverse-Electron-Demand Diels-Alder Reaction. Chembiochem.

[179] Yeleswarapu S., Chameettachal S., Bera A.K., Pati F. (2022). Smooth muscle matrix bioink promotes myogenic differentiation of encapsulated adipose-derived stem cells. J. Biomed. Mater. Res. Part A.

[180] Mohiuddin O.A., O’Donnell B.T., Poche J.N., Iftikhar R., Wise R.M., Motherwell J.M., Campbell B., Savkovic S.D., Bunnell B.A., Hayes D.J. (2019). Human adipose-derived hydrogel characterization based on in vitro ASC biocompatibility and differentiation. Stem Cells Int..

[181] Shridhar A., Amsden B.G., Gillies E.R., Flynn L.E. (2019). Investigating the Effects of Tissue-Specific Extracellular Matrix on the Adipogenic and Osteogenic Differentiation of Human Adipose-Derived Stromal Cells Within Composite Hydrogel Scaffolds. Front. Bioeng. Biotechnol..

[182] Coronado R.E., Somaraki-Cormier M., Ong J.L., Halff G.A. (2019). Hepatocyte-like cells derived from human amniotic epithelial, bone marrow, and adipose stromal cells display enhanced functionality when cultured on decellularized liver substrate. Stem Cell Res..

[183] Asadi M., Lotfi H., Salehi R., Mehdipour A., Zarghami N., Akbarzadeh A., Alizadeh E. (2021). Hepatic cell-sheet fabrication of differentiated mesenchymal stem cells using decellularized extracellular matrix and thermoresponsive polymer. Biomed. Pharmacother..

[184] Guneta V., Zhou Z., Tan N.S., Sugii S., Wong M.T.C., Choong C. (2018). Recellularization of decellularized adipose tissue-derived stem cells: Role of the cell-secreted extracellular matrix in cellular differentiation. Biomater. Sci..

[185] Zhang Z., Qu R., Fan T., Ouyang J., Lu F., Dai J. (2019). Stepwise Adipogenesis of Decellularized Cellular Extracellular Matrix Regulates Adipose Tissue-Derived Stem Cell Migration and Differentiation. Stem Cells Int..

[186] Blum J.C., Schenck T.L., Birt A., Giunta R.E., Wiggenhauser P.S. (2021). Artificial decellularized extracellular matrix improves the regenerative capacity of adipose tissue derived stem cells on 3D printed polycaprolactone scaffolds. J. Tissue Eng..

[187] Qian Y., Chen H., Pan T., Li T., Zhang Z., Lv X., Wang J., Ji Z., He Y., Li L. (2021). Autologous decellularized extracellular matrix promotes adipogenic differentiation of adipose derived stem cells in low serum culture system by regulating the ERK1/2-PPARγ pathway. Adipocyte.

